# Microwave-assisted H_2_SO_4_ and H_2_SO_4_/HNO_3_ hydrolysis for process-intensified cellulose nanocrystal production with structural evolution and surface functionalization

**DOI:** 10.1039/d6ra05553b

**Published:** 2026-09-16

**Authors:** Chakkaphan Pho-ngernngam, Ekarat Detsri, Piyathida Thaipukdee, Nawasit Chotsaeng, Suparat Rukchonlatee, Arjnarong Mathaweesansurn

**Affiliations:** a Department of Chemistry, School of Science, King Mongkut's Institute of Technology Ladkrabang Bangkok 10520 Thailand Ekarat.de@Kmitl.ac.th 67056007@Kmitl.ac.th Piyathida.tpd@Gmail.com Nawasit.ch@Kmitl.ac.th Suparat.ru@Kmitl.ac.th Arjnarong.ma@Kmitl.ac.th; b Integrated Applied Chemistry Research Unit, School of Science, King Mongkut's Institute of Technology Ladkrabang Bangkok 10520 Thailand; c Advanced Pure and Applied Chemistry Research Unit, School of Science, King Mongkut's Institute of Technology Ladkrabang Bangkok 10520 Thailand; d Polymer Synthesis and Functional Materials Research Unit, School of Science, King Mongkut's Institute of Technology Ladkrabang Bangkok Thailand; e Applied Analytical Chemistry Research Unit, School of Science, King Mongkut's Institute of Technology Ladkrabang Bangkok 10520 Thailand

## Abstract

Microwave-assisted acid hydrolysis was developed as an intensified strategy for producing cellulose nanocrystals (CNCs) from cotton-derived cellulose. Single-acid H_2_SO_4_ and mixed-acid H_2_SO_4_/HNO_3_ hydrolysis systems were systematically investigated under controlled microwave irradiation to elucidate the role of acid composition in governing CNC formation, crystalline evolution, surface functionality, thermal stability, and colloidal behavior. Microwave-induced volumetric heating promoted rapid acid–cellulose interactions and efficient cleavage of amorphous cellulose domains, enabling CNC isolation within markedly shortened reaction times compared with conventional thermal hydrolysis. Under optimized conditions, microwave-assisted H_2_SO_4_ hydrolysis produced CNCs with a yield of 49.0%, crystallinity index of 86.4%, aspect ratio of 5.3 ± 2.7, and sulfate half-ester groups (–OSO_3_^−^) content of 0.14 mmol g^−1^. In comparison, microwave-assisted H_2_SO_4_/HNO_3_ hydrolysis yielded CNCs with a comparable yield of 47.7%, higher crystallinity index of 89.2%, aspect ratio of 7.2 ± 2.7, and –OSO_3_^−^ group content of 0.13 mmol g^−1^. The mixed-acid route further introduced carboxyl/carboxylate (–COOH/–COO^−^) functionalities through limited HNO_3_-assisted oxidation, as evidenced by FT-IR, solid-state ^13^C NMR, XPS, and zeta potential analyses. Consequently, the mixed-acid CNCs exhibited the most negative zeta potential (−42.4 ± 0.9 mV) and the highest thermal stability, with a *T*_5%_ of 285.7 °C and a major DTG peak at 355.4 °C. Compared with conventional H_2_SO_4_ hydrolysis, microwave-assisted hydrolysis improved CNC recovery while reducing acid concentration and processing time. These findings demonstrate that microwave-assisted mixed-acid hydrolysis provides a controllable and efficient route for producing CNCs with tunable crystallinity, surface functionality, colloidal stability, and thermal performance.

## Abstract 2

Microwave-assisted acid hydrolysis was developed as an intensified strategy for producing cellulose nanocrystals (CNCs) from cotton-derived cellulose.

## Graphical abstract

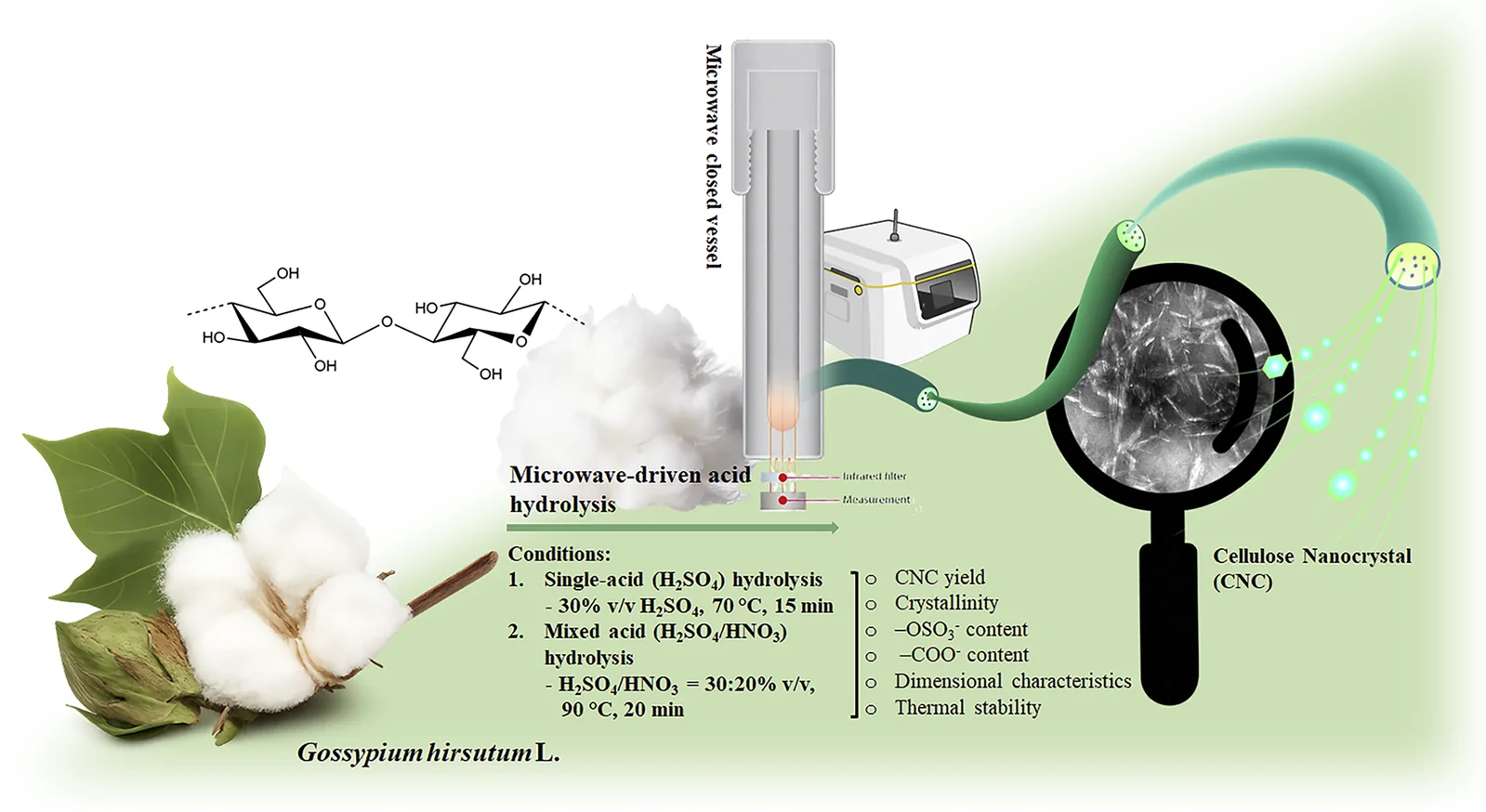

## 1. Introduction

Cellulose nanocrystals (CNCs) have attracted considerable attention as renewable bio-based nanomaterials owing to their high crystallinity,^1,2^ nanoscale rod-like morphology, low density (1.5–1.6 g cm^−3^),^3,4^ biodegradability,^5,6^ and excellent mechanical properties, with reported tensile strength and elastic modulus values of 10 ± 3.02 GPa and 150 ± 11.25 GPa,^7^ respectively. Derived from the crystalline domains of native cellulose, CNCs possess a high aspect ratio and abundant surface hydroxyl groups, which provide strong reinforcement capability^8,9^ and enable further surface modification.^10^ These characteristics make CNCs promising materials for applications in polymer nanocomposites,^10–12^ rheology modifiers,^13^ coatings,^14^ packaging materials,^15,16^ membranes,^17,18^ sensors,^19–21^ biomedical scaffolds,^22^ and drug-delivery systems.^23,24^ In addition, their renewable origin, non-toxicity, and compatibility with aqueous processing further highlight their potential for sustainable material development. The increasing interest in CNCs therefore aligns with global efforts to develop circular, sustainable, and carbon-neutral material technologies.

Conventionally, CNCs are prepared by acid hydrolysis of lignocellulosic or cellulose-rich feedstocks, in which amorphous cellulose regions are selectively removed while highly crystalline domains are retained. Among the available methods, sulfuric acid (H_2_SO_4_)^25–27^ hydrolysis is widely used because it introduces sulfate half-ester groups (−OSO_3_^−^), which enhance colloidal stability. Other preparation routes include hydrochloric acid (HCl) hydrolysis,^28,29^ mixed-acid hydrolysis,^30,31^ enzymatic treatment,^32^ high-pressure homogenization,^33^ and oxidative processes such as TEMPO-mediated oxidation.^34^ A summary of representative CNC preparation methods, including their advantages, limitations, and key product characteristics, is provided in Table S1. Despite their effectiveness, conventional thermal hydrolysis methods generally require long reaction times, high acid consumption, and intensive washing or post-treatment steps, which limit their scalability and sustainability.^35,36^ Therefore, process-intensified strategies that enable rapid and uniform heating, improved hydrolysis efficiency, and reduced chemical consumption are needed for more sustainable CNC production.

Among these emerging approaches, microwave-assisted acid hydrolysis has shown significant promise, offering rapid volumetric heating (2.0 × 10^7^ W m^−3^), shortened reaction times (30 min), and improved control over nanoscale structural evolution.^37,38^ Unlike conventional conductive heating, microwave irradiation generates heat directly within the reaction medium through dipolar rotation and ionic conduction,^39^ enabling rapid energy transfer and improved thermal homogeneity. This heating mechanism can accelerate the cleavage of amorphous cellulose regions while reducing acid consumption and minimizing excessive cellulose degradation.^40^ When combined with tailored acid systems, such as mixed H_2_SO_4_/HNO_3_, microwave-assisted hydrolysis may provide additional control over CNC surface chemistry, crystallinity, morphology, and colloidal stability. However, the effects of microwave irradiation on different acid systems remain insufficiently understood. Most previous studies have focused mainly on H_2_SO_4_-based hydrolysis and emphasized reaction acceleration, while the potential of mixed-acid systems to regulate sulfate/carboxyl functionality, particle dimensions, crystallinity, and thermal behavior has received less attention. This knowledge gap limits the rational design of greener and more efficient hydrolysis pathways for CNC production.

To address these knowledge gaps and unresolved limitations, the present work systematically investigates the influence of microwave-assisted acid hydrolysis on the structural development of cellulose nanocrystals. This study focuses on the roles of acid composition and microwave-assisted reaction conditions in controlling CNC morphology, crystallinity, and surface chemistry. Specifically, single-acid (H_2_SO_4_) and mixed-acid (H_2_SO_4_/HNO_3_) hydrolysis systems are compared under controlled microwave irradiation to clarify their hydrolysis behavior and surface-functionalization pathways. By integrating structural characterization with process analysis, this work aims to develop a more energy-efficient and tunable CNC production strategy with reduced chemical consumption, improved environmental performance, and potential industrial relevance.

## 2. Experimental

### 2.1 Chemicals and reagents

#### 2.1.1 Chemicals and reagents

Cotton cellulose fibers, produced from 100% purified cotton, were purchased from Evergreen Co., Ltd (Japan) and used as the primary cellulose source. Concentrated sulfuric acid (96% H_2_SO_4_, Carlo Erba Co., Ltd, Italy) and nitric acid (65% HNO_3_, Thermo Scientific Chemicals Co., Ltd, USA) were utilized as the acid hydrolytic. Sodium hydroxide (NaOH, ≥97%), sodium bicarbonate (NaHCO_3_), hydrochloric acid (HCl, 37%), ethanol (C_2_H_5_OH, 99.5%), and acetone (CH_3_)_2_CO were also acquired from Carlo Erba Co., Ltd, Italy. Regenerated cellulose dialysis tubing (molecular weight cut-off, MWCO 14 kDa, Sigma-Aldrich Co., Ltd, USA) was used for removing residual acids and low-molecular-weight species during sample purification. Deionized (DI) water (resistivity ≥ 18.2 MΩ cm) was used in all experiments. All chemicals were of analytical grade and were used without further purification.

#### 2.1.2 Instrumentation

The surface morphology and dimensional characteristics of CNCs were examined using a high-resolution transmission electron microscope (HR-TEM, JEM-2010, JEOL Co., Ltd, Japan). Elemental composition was analyzed using energy-dispersive X-ray spectroscopy (EDS, IXRF system, EDS2006 550i Analyzer). The elemental composition, including carbon, hydrogen, nitrogen, and sulfur contents, was determined using a CHNS elemental analyzer (FlashSmart™ Elemental Analyzer, USA). The crystallinity and crystal structure of CNCs were characterized using an X-ray diffractometer (XRD, Rigaku, Japan) equipped with a CuKα radiation source (*λ* = 0.15406 nm) over a 2*θ* range of 10–40°. Functional groups and surface chemical bonding were identified by Fourier transform infrared spectroscopy (ATR-FTIR, IRTracer-100, Shimadzu Co., Ltd, Kyoto, Japan) over the spectral range of 4000–700 cm^−1^. The chemical structure and cellulose integrity were further examined by solid-state ^13^C nuclear magnetic resonance (^13^C NMR) spectroscopy (JEOL, JNM-ECZ-400R/S1), providing insights into carbon environments and crystallinity-related resonances. The surface chemical states and elemental environments of CNCs were additionally analyzed using X-ray photoelectron spectroscopy (XPS, AXIS Ultra DLD, KRATOS Analytical Co., Ltd, UK). Thermal stability and decomposition profiles were evaluated by thermogravimetric analysis (TGA, TGA/DSC^3+^ STARe system, Mettler Toledo, Switzerland) under a nitrogen atmosphere. Surface charge and colloidal stability were assessed using a zeta potential analyzer (Zetasizer Nano ZS, Malvern, UK). Furthermore, a field-emission scanning electron microscope (FE-SEM, SU5000, Hitachi, Japan) was employed to observe the surface morphology and deposition behavior of CNCs on the cotton substrate.

### 2.2 Microwave-driven acid hydrolysis of cellulose nanocrystal

**Fig. 1:**
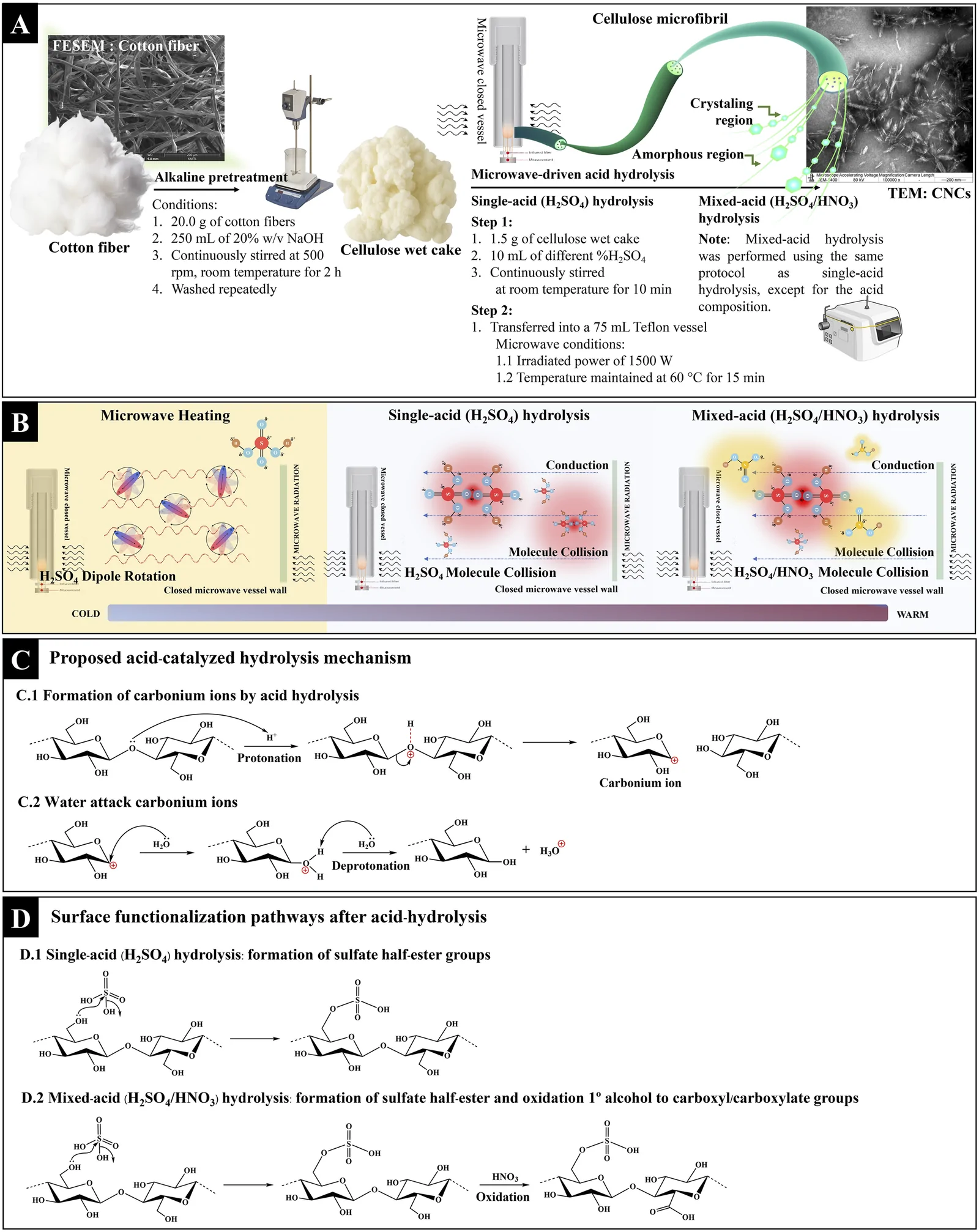
Schematic diagram of (A) CNC synthesis conditions, (B) microwave heating interaction, (C) and (D) microwave heating mechanism of H_2_SO_4_, and microwave heating mechanism of H_2_SO_4_/HNO_3_.

Cotton cellulose fibers were pretreated under alkaline conditions (20% w/v NaOH) to remove hemicelluloses (*e.g.*, xylans, arabinoxylans, glucomannans), pectins (*e.g.*, pectic acids and galacturonic residues), and natural pigments (*e.g.*, carotenoids and flavonoids). Typically, 20.0 g of cotton fibers were dispersed in 250 mL of 20% w/v NaOH solution and continuously stirred at 500 rpm, room temperature for 2 h. After alkaline pretreatment, the suspension was cooled to room temperature (25 °C) and filtered with Whatman no. 1-filter paper (CHM™, Barcelona, Spain) under vacuum pump (V-180, Buchi, Switzerland). The treated fibers were washed repeatedly with deionized water until the filtrate reached neutral pH, indicating the complete removal of residual alkali and soluble impurities. The purified cellulose fibers were then collected as a wet cake and used directly as the starting material for the subsequent acid hydrolysis to produce cellulose nanocrystals (CNCs) (Fig. 1A).

#### 2.2.1 Single-acid (H_2_SO_4_) hydrolysis

Single-acid hydrolysis of alkali-pretreated cotton cellulose fibers was carried out using sulfuric acid (96% H_2_SO_4_) under microwave irradiation. A wet cake of pretreated cellulose corresponding to 1.5 g was dispersed in 10 mL of H_2_SO_4_ solution at different concentrations. The suspension was magnetically stirred at room temperature for 10 min to obtain a homogeneous dispersion and then transferred into a 75 mL Teflon (polytetrafluoroethylene, PTFE) vessel suitable for microwave processing. The vessel was placed in a microwave reactor (Titan MPS microwave sample preparation system, PerkinElmer Co., Ltd (USA)) operating at a microwave frequency of 2.45 GHz and irradiated at a microwave power of 1500 W while the reaction temperature maintained at 60 °C for 15 min. After hydrolysis, the hot reaction mixture was immediately quenched by pouring it into 400 mL of ice-cooled deionized water under vigorous stirring to stop further acid-hydrolysis. The collected CNC fraction was redispersed in deionized water and centrifuged repeatedly until the supernatant reached a pH of 2–3. The supernatant was collected and subsequently subjected to dialysis using regenerated cellulose dialysis tubing (MWCO 14 kDa) against D.I. water until the conductivity of the dialysate decreased to below 20 µS cm^−1^ (S23030019034, Mettler Toledo, Switzerland). Then the resulting suspension was frozen at −80 °C and freeze-dried for 48 h to obtain the to obtain a dry white CNC powder suitable for subsequent physicochemical characterization.

To investigate the effects of process conditions on the structural and physicochemical properties of the resulting CNCs, various hydrolysis parameters were systematically varied, including sulfuric acid concentrations of 20, 30, 40, and 50% v/v, reaction temperatures of 60, 70, 80, and 90 °C, and hydrolysis times of 10, 15, 20, and 25 min. A microwave irradiation power of 1500 W was applied to achieve rapid and controlled heating during acid hydrolysis. In each experiment, only one parameter was varied while all other conditions were kept constant to clearly evaluate its individual effect. This one-factor-at-a-time (OFAT) approach was selected as an initial process-screening strategy to maintain experimental feasibility and enable direct comparison of the effects of each hydrolysis parameter on CNC yield, crystallinity, morphology, and surface functionality. Each experimental condition was performed in triplicate to ensure reproducibility and statistical reliability of the results.

#### 2.2.2 Mixed acid (H_2_SO_4_/HNO_3_) hydrolysis

The experimental procedure for mixed-acid (H_2_SO_4_/HNO_3_) hydrolysis was identical to that described in Section 2.2.1 for single-acid (H_2_SO_4_) hydrolysis, except that the hydrolyzing medium was replaced with a mixed-acid system. In this set of experiments, the concentration of H_2_SO_4_ was fixed at 30% v/v, while the concentration of HNO_3_ was varied within the range of 5–30% v/v. To further examine the effects of process variables on the structural and physicochemical properties of the resulting CNCs, several hydrolysis parameters were additionally varied, including reaction temperatures of 60–90 °C, and hydrolysis times of 10–25 min. The reactor automatically adjusted the microwave power up to 1500 W to maintain the preset hydrolysis temperature.

### 2.3 Conventional heat H_2_SO_4_ hydrolysis

Alkali-pretreated cotton cellulose fibers (1.5 g) were dispersed in 50% v/v H_2_SO_4_ solution and continuously stirred at 50 °C for 2 h. After hydrolysis, the reaction was immediately quenched by adding excess cold deionized water. The resulting suspension was centrifuged and repeatedly washed with deionized water to remove residual acid. The suspension was then dialyzed (cellulose dialysis tubing, MWCO 14 kDa) against DI water until a neutral pH was reached, followed by ultrasonication and freeze-drying to obtain CNC powder.

### 2.4 Data analysis and property calculations

Data analysis and property calculations were conducted to compare CNCs obtained from single-acid (H_2_SO_4_) and mixed-acid (H_2_SO_4_/HNO_3_) hydrolysis. The obtained CNCs were systematically evaluated to compare the effects of single-acid H_2_SO_4_ and mixed-acid H_2_SO_4_/HNO_3_ hydrolysis on CNC yield, crystallinity index, surface functionality, dimensional characteristics, and thermal stability.

#### 2.4.1 CNC yield

The yield of cellulose nanocrystals (CNCs) was determined gravimetrically based on the mass of freeze-dried CNCs obtained from a known amount of alkali-pretreated cotton cellulose. For each hydrolysis condition, a defined dry mass of pretreated cellulose (1.5 g) was subjected to single-acid or mixed-acid hydrolysis, followed by quenching, purification, and freeze-drying as described above. The resulting CNC powder was dried to constant weight in a vacuum desiccator and weighed using an analytical balance (±0.1 mg). The CNC yield was calculated according to eqn (1):1
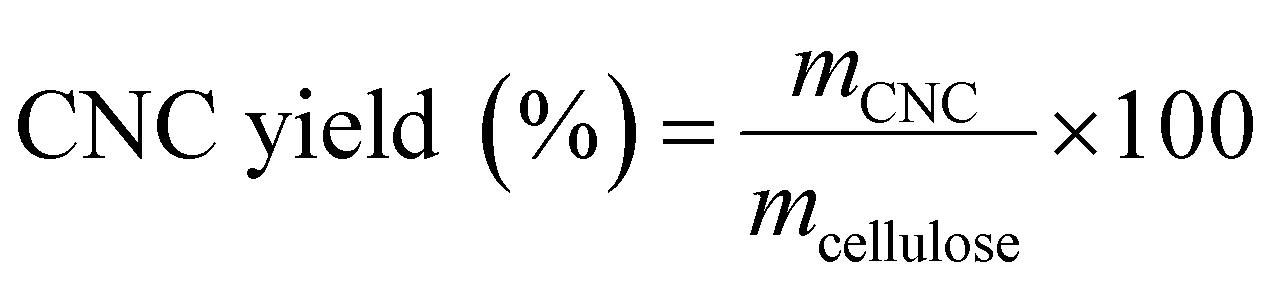
where *m*_CNC_ is the mass of the dried CNC powder (g) and *m*_cellulose_ is the initial dry mass of alkali-pretreated cellulose used for hydrolysis (g).

All yield measurements were performed in five replicates for each condition, and the results are reported as mean ± standard deviation.

#### 2.4.2 Crystallinity

The crystallinity index (CrI) of the CNC samples was determined from X-ray diffraction (XRD) patterns using the peak-deconvolution method, in which the peaks were fitted with Gaussian functions using Origin™ software. The freeze-dried CNC powder was gently ground and packed into the sample holder, and XRD measurements were performed using CuKα radiation (*λ* = 0.15406 nm) over a 2*θ* range of 10–40°. The CrI was calculated according to eqn (2):2
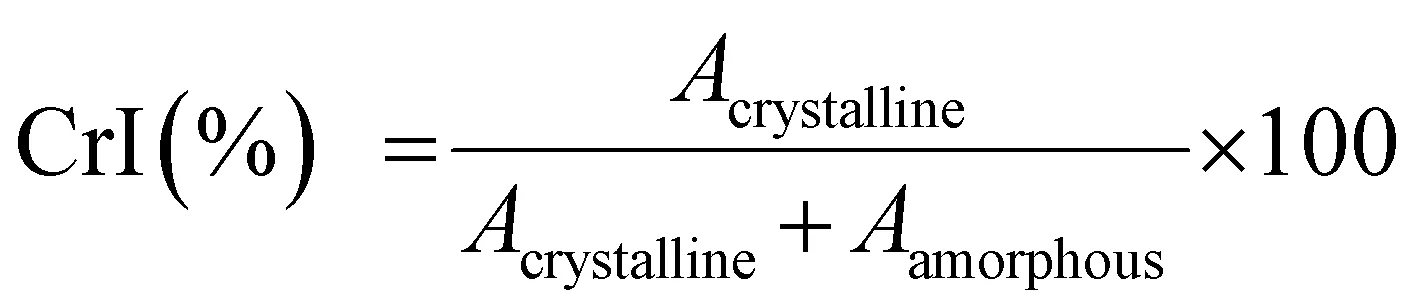
where *A*_crystalline_ and *A*_amorphous_ are the integrated areas of all crystalline and amorphous regions, respectively, obtained from XRD peak deconvolution.

All crystallinity measurements were performed in five replicates, and the results are reported as mean ± standard deviation.

#### 2.4.3 Sulfate half-ester group and carboxyl content

The surface chemical functionalities of CNCs, particularly sulfate half-ester groups (–OSO_3_^−^) and carboxyl/carboxylate groups (–COOH/–COO^−^), were evaluated using CHNS elemental analysis and X-ray photoelectron spectroscopy (XPS). The sulfate group content was estimated from the sulfur content obtained by CHNS analysis, assuming one sulfur atom per sulfate half-ester group. The sulfate content was calculated using the following eqn (3):3
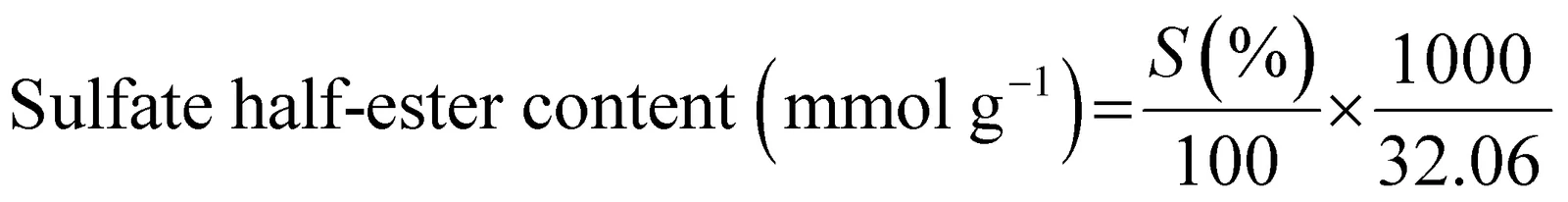
where *S* (%) is the sulfur content obtained from CHNS analysis and 32.06 g mol^−1^ is the atomic mass of sulfur.

Carboxyl/carboxylate functionalities were evaluated from the high-resolution C 1s spectra obtained by XPS. The O–C=O component was used as an indicator of carboxyl/carboxylate groups on the CNC surface. The relative O–C=O contribution was calculated according to eqn (4):4
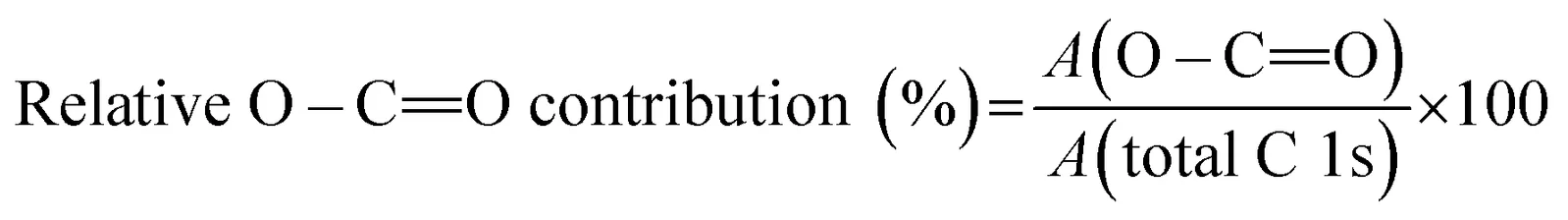
where *A*(O–C=O) is the fitted peak area of the O–C=O component and *A* (total C 1s) is the total fitted area of all C 1s components.

#### 2.4.4 Dimensional characteristics

The dimensional characteristics of the CNCs, including particle length (*L*), diameter (*D*), and aspect ratio (*L*/*D*), were determined from transmission electron microscopy (TEM) images. The freeze-dried CNC powder was dispersed in deionized water and ultrasonicated (high-power ultrasonicate, VCX 130, Vibra-Cell™, USA) for 10 min to obtain a well-dispersed suspension. A drop of the diluted suspension was deposited onto a carbon-coated copper grid, negatively stained with 1 wt% uranyl acetate (C_4_H_6_O_6_U) to enhance image contrast, and air-dried prior to imaging. TEM micrographs were acquired at appropriate magnifications, and particle dimensions were measured using ImageJ software. For each sample, at least 60 individual nanocrystals were randomly selected and measured to obtain statistically representative values. The aspect ratio (*L*/*D*) was calculated using eqn (5):5
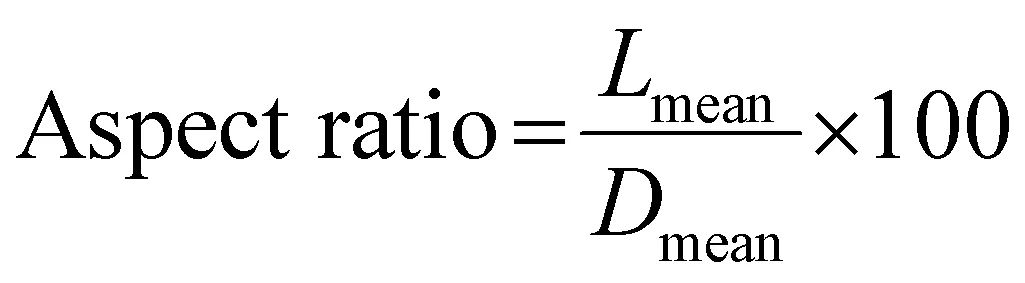
where *L*_mean_ and *D*_mean_ represent the average particle length and diameter, respectively. Size-distribution histograms were constructed to evaluate particle uniformity and polydispersity.

#### 2.4.5 Thermal stability

The thermal stability of the obtained CNCs was evaluated using thermogravimetric analysis (TGA). 10 mg of freeze-dried CNC powder was placed in a platinum sample pan and heated from 25 to 900 °C at a constant heating rate of 20 °C min^−1^ under a nitrogen atmosphere (20 mL min^−1^). The TGA and derivative thermogravimetric (DTG) curves were recorded to determine the onset decomposition temperature (*T*_onset_), maximum degradation temperature (*T*_max_), and char residue at 900 °C.

#### 2.4.6 Activation energy

The apparent activation energy (*E*_a_) of thermal degradation was determined from TGA data using the Horowitz–Metzger method.^41^ The Horowitz–Metzger equation is expressed as:6
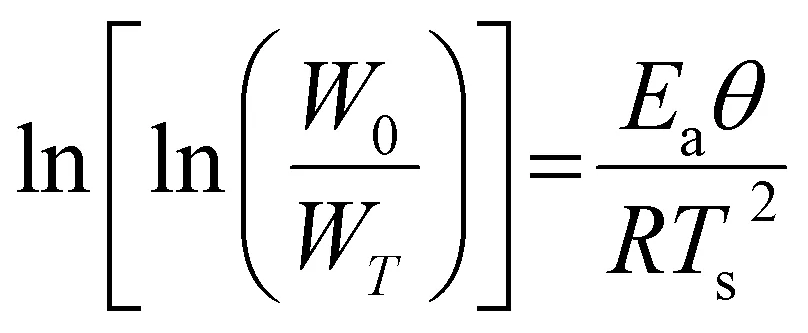
where *E*_a_ is the activation energy, *R* is the gas constant (8.314 J mol^−1^ K^−1^), *T*_s_ the maximum decomposition temperature obtained from the DTG peak in Kelvin, *θ* = *T* − *T*_s_, *W*_0_ is the initial mass at the beginning of the selected degradation stage, *W*_*T*_ is the residual mass at temperature *T*. For each degradation stage, a linear plot of ln[ln(*W*_0_/(*W*_*T*_)] *versus θ* was constructed. The activation energy was calculated from the slope of the plot using:

7 *E*_a_ = slope × *RT*_s_^2^

## 3. Results and discussion

### 3.1 CNC isolation and optimization

**Fig. 2:**
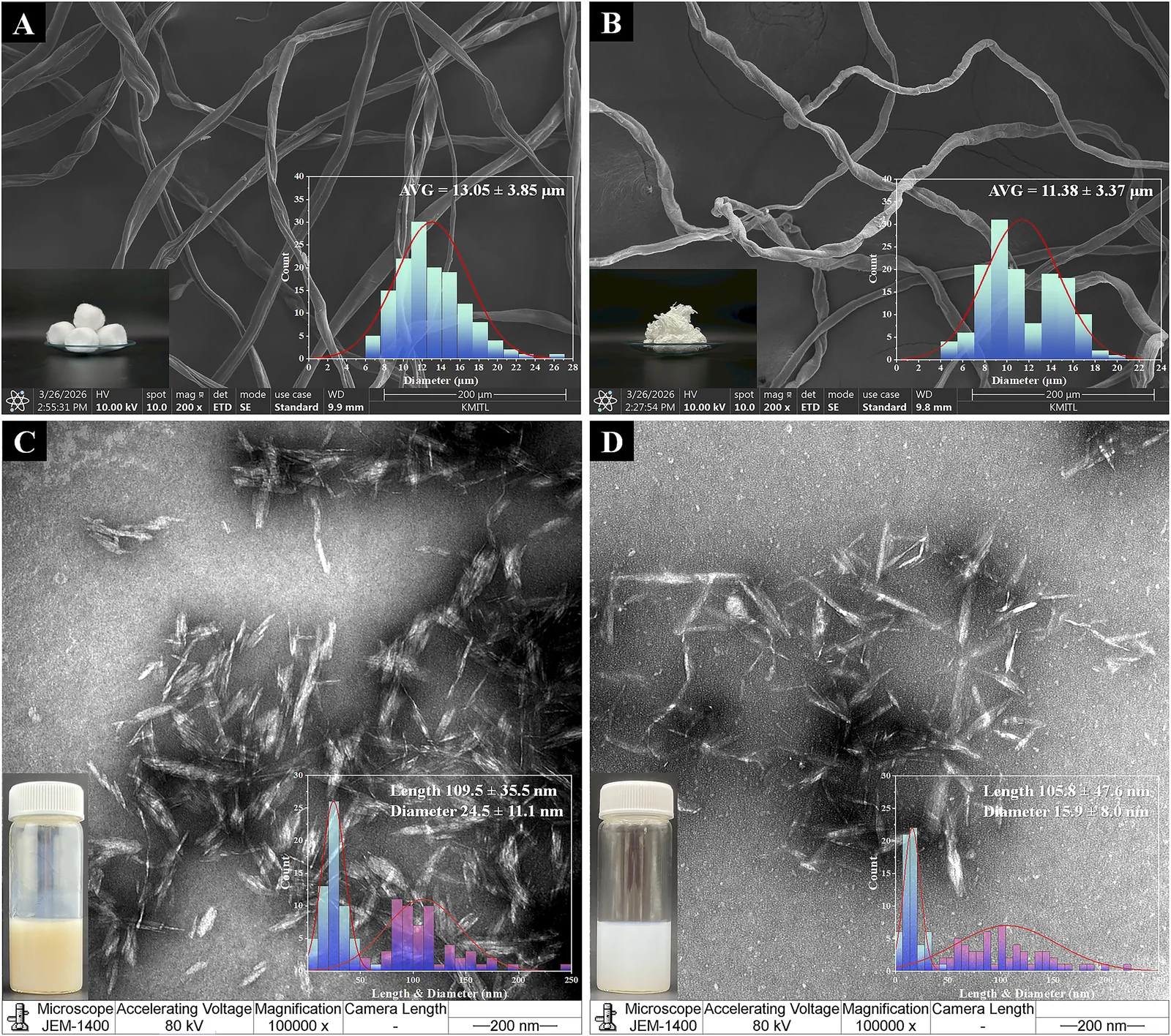
FESEM images of (A) cotton and (B) alkali-treated cotton, and TEM images of CNCs prepared by (C) microwave-assisted H_2_SO_4_ hydrolysis and (D) microwave-assisted H_2_SO_4_/HNO_3_ hydrolysis. Insets show diameter-distribution histograms and digital photographs of the corresponding samples.

CNCs were isolated from cotton-derived cellulose fibers (CF) through microwave-assisted acid hydrolysis using single-acid H_2_SO_4_ and mixed-acid H_2_SO_4_/HNO_3_ systems. As illustrated in Fig. 1A, the raw cotton fibers were first subjected to alkaline pretreatment (NaOH, 20% w/v) to remove non-cellulosic components^42^ and obtain purified cellulose wet cake. The morphological evolution of cotton fibers before and after alkaline pretreatment was examined by FESEM (Fig. 2A and B). The pristine cotton fibers exhibited long, twisted ribbon-like microfibers with relatively smooth surfaces and an average diameter of 13.05 ± 3.85 µm (Fig. 2A). Following NaOH pretreatment, the fibers became looser, rougher, and partially fibrillated (Fig. 2B), reflecting the removal of surface impurities and non-cellulosic constituents. Subsequently, the cellulose wet cake was hydrolyzed in a sealed microwave vessel under controlled irradiation at 2.45 GHz, using H_2_SO_4_ or mixed H_2_SO_4_/HNO_3_ as the hydrolyzing medium. Microwave-induced volumetric heating enabled rapid temperature elevation and uniform energy distribution within the acidic suspension, thereby enhancing acid diffusion and heat transfer. Under the optimized hydrolysis conditions, this intensified heating environment accelerated^43^ the selective cleavage of β(1→4)-glycosidic bonds within the amorphous regions of cellulose, while preserving the more ordered crystalline domains. As depicted in Fig. 1B, the combined effects of dipolar polarization and ionic conduction in the acid medium promoted efficient acid–cellulose interactions, leading to the progressive disintegration of cellulose fibers into rod-like CNCs.

#### 3.1.1 Single-acid (H_2_SO_4_) hydrolysis

Under microwave irradiation, hydrolysis of cellulose in H_2_SO_4_ exhibits markedly different physicochemical behavior from aqueous systems,^44^ owing to the strong interaction between microwave energy and the ionic acidic medium. H_2_SO_4_ possesses a high dielectric constant (*ε*′ = 29.5),^45^ a large dielectric loss factor (*ε*″ = 192.2),^45^ and strong ionic conductivity (131.4 mS cm^−1^ at 50 °C)^46^ arising from mobile charge carriers, including H^+^, HSO_4_^−^, and SO_4_^2−^. This intensified heating environment directly accelerates the H_2_SO_4_–cellulose reaction mechanism. H_2_SO_4_ first protonates the glycosidic oxygen of the β(1→4)-linkage, weakening the C–O–C bond and inducing glycosidic bond cleavage to generate carbonium ion intermediates. These intermediates are subsequently attacked by water (H_2_O) molecules (Fig. 1C), followed by deprotonation, producing shorter cellulose chains with new hydroxyl termini. Meanwhile, partial esterification of cellulose surface hydroxyl groups by H_2_SO_4_ generates sulfate half-ester groups (Fig. 1D.1), which impart negative surface charges and enhance colloidal stability of the obtained CNCs. Since amorphous domains are more accessible to acid attack than crystalline regions, hydrolysis occurs preferentially in amorphous cellulose, leaving crystalline segments as cellulose nanocrystals.

#### 3.1.2 Effect of H_2_SO_4_ concentration

**Fig. 3:**
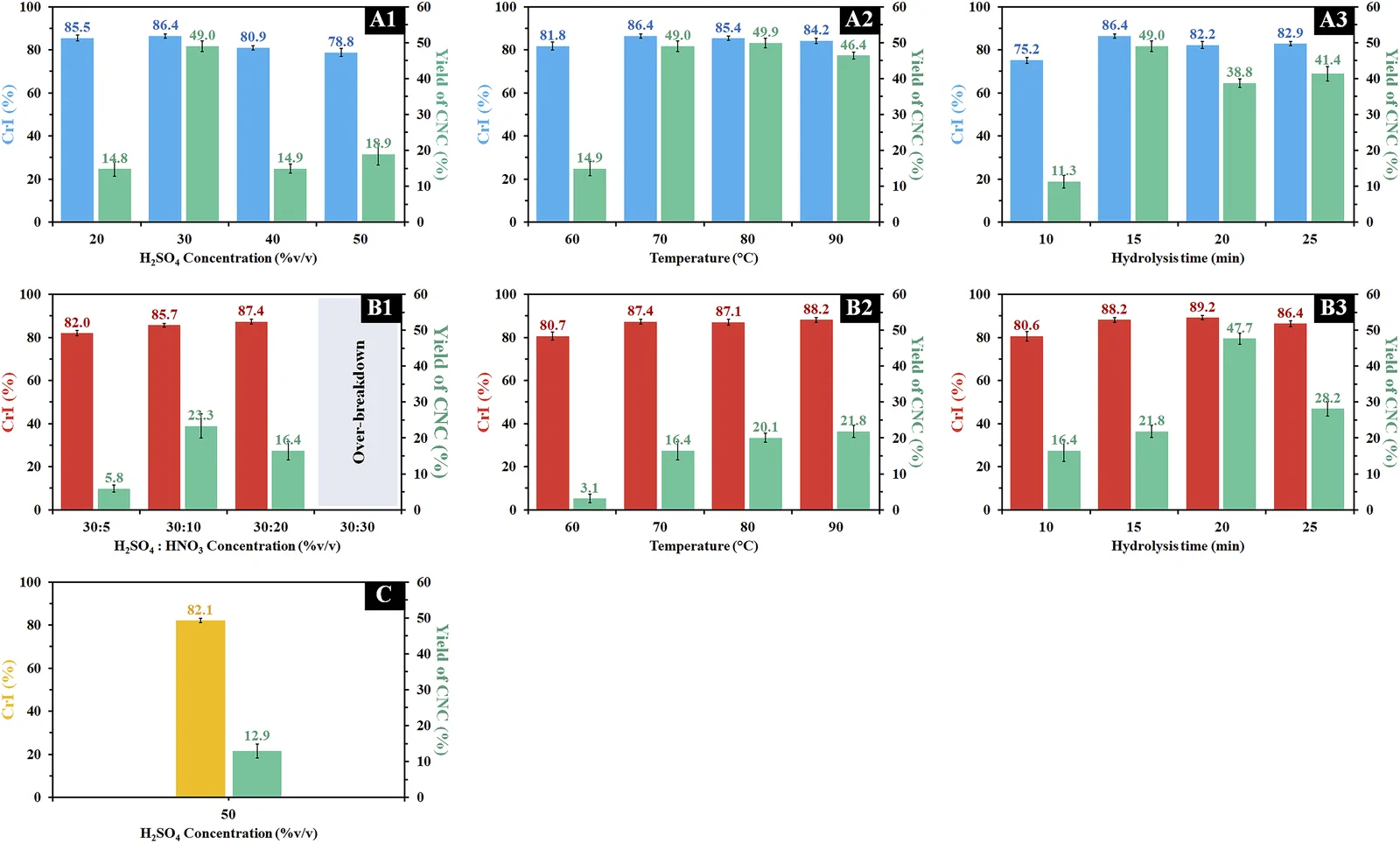
Optimization of CNC preparation conditions and their effects on the crystallinity index (CrI) and CNC yield: (A) microwave-assisted H_2_SO_4_ hydrolysis under different (A1) H_2_SO_4_ concentrations (20–50% v/v), (A2) hydrolysis temperatures (60–90 °C), and (A3) hydrolysis times (10–25 min), (B) microwave-assisted H_2_SO_4_/HNO_3_ hydrolysis under different (B1) H_2_SO_4_/HNO_3_ concentration ratios (30 : 5, 30 : 10, 30 : 20, and 30 : 30% v/v), (B2) hydrolysis temperatures (60–90 °C), and (B3) hydrolysis times (10–25 min), and (C) conventional H_2_SO_4_ hydrolysis.

**Fig. 4:**
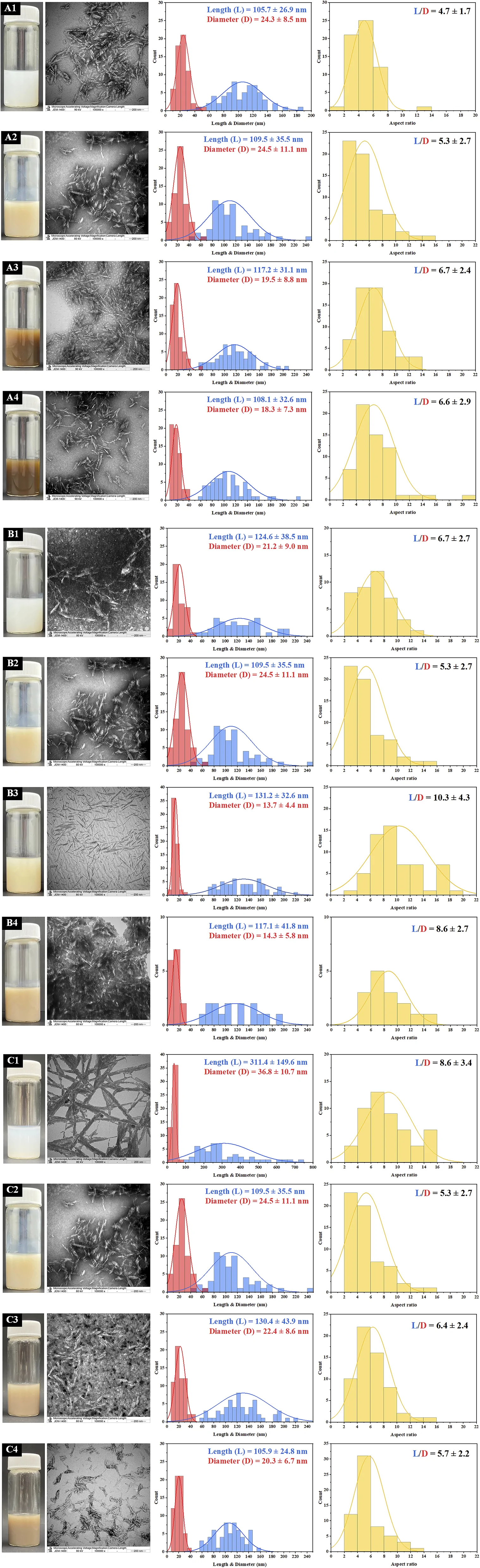
TEM images of CNC prepared by microwave-assisted H_2_SO_4_ hydrolysis, with inset digital photographs, diameter-distribution histograms, and aspect ratio analysis under different H_2_SO_4_ concentrations: (A1) 20% v/v, (A2) 30% v/v, (A3) 40% v/v, and (A4) 50% v/v. TEM images of CNC prepared by microwave-assisted H_2_SO_4_ hydrolysis, with inset digital photographs, diameter-distribution histograms, and aspect ratio analysis under different hydrolysis temperature: (B1) 60 °C, (B2) 70 °C, (B3) 80 °C, and (B4) 90 °C. TEM images of CNC prepared by microwave-assisted H_2_SO_4_ hydrolysis, with inset digital photographs, diameter-distribution histograms, and aspect ratio analysis under different hydrolysis times: (C1) 10 min, (C2) 15 min, (C3) 20 min, and (C4) 25 min.

The influence of H_2_SO_4_ concentration on CNC production during microwave-assisted hydrolysis is presented in Fig. 3A1. At H_2_SO_4_ concentrations of 20, 30, 40, and 50% v/v, the CNC yields were 14.8, 49.0, 14.9, and 18.9%, respectively, while the corresponding CrI values were 85.5, 86.4, 80.9, and 78.8%, respectively. The low yield at 20% H_2_SO_4_ indicates insufficient acid strength for effective disintegration of cellulose fibers into nanoscale crystalline fragments. Increasing the H_2_SO_4_ concentration to 30% markedly enhanced the CNC yield while maintaining a high CrI, suggesting effective hydrolysis of amorphous cellulose domains without significant disruption of crystalline regions. In contrast, higher acid concentrations of 40 and 50% v/v reduced both yield and CrI, likely due to excessive acid-catalyzed hydrolysis, over-depolymerization, dissolution of hydrolyzed cellulose fragments, and partial erosion of crystalline domains. TEM analysis further confirmed the formation of rod-like CNCs under all acid concentrations (Fig. 4A1–A4). The CNCs prepared using 20, 30, 40, and 50% H_2_SO_4_ exhibited average lengths of 105.7 ± 26.9, 109.5 ± 35.5, 117.2 ± 31.1, and 108.1 ± 32.6 nm, respectively, with corresponding diameters of 24.3 ± 8.5, 24.5 ± 11.1, 19.5 ± 8.8, and 18.3 ± 7.3 nm. The corresponding aspect ratio was 4.7 ± 1.7, 5.3 ± 2.7, 6.7 ± 2.4, and 6.6 ± 2.9, respectively. Although higher H_2_SO_4_ concentration produced slightly thinner CNCs with higher aspect ratio, the accompanying reductions in yield and CrI indicate excessive hydrolysis and partial degradation of crystalline cellulose. Therefore, 30% v/v H_2_SO_4_ was selected as the optimal concentration for microwave-assisted single-acid hydrolysis, providing the most favorable balance among CNC yield, crystallinity preservation, and rod-like nanoscale morphology.

#### 3.1.3 Effect of reaction temperature

The effect of reaction temperature on CNC production during microwave-assisted H_2_SO_4_ hydrolysis is shown in Fig. 3A2. Increasing the temperature from 60 to 80 °C enhanced the CNC yield from 14.9 to 49.9%, while the CrI increased from 81.8 to 86.4%, indicating more effective removal of amorphous cellulose regions. However, further increasing the temperature to 90 °C reduced the yield to 46.4%, respectively, with a slight decrease in CrI to 84.2%. This decline is likely due to excessive hydrolysis and partial degradation of cellulose at elevated temperatures. TEM analysis further confirmed the temperature-dependent morphology of the obtained CNCs (Fig. 4B1–B4). The CNCs prepared at 60, 70, 80, and 90 °C exhibited average lengths of 124.6 ± 38.5, 109.5 ± 35.5, 131.2 ± 32.6, and 117.1 ± 41.8 nm, respectively, with corresponding diameters of 21.2 ± 9.0, 24.5 ± 11.1, 13.7 ± 4.4, and 14.3 ± 5.8 nm. The corresponding aspect ratios were 6.7 ± 2.7, 5.3 ± 2.7, 10.3 ± 4.3, and 8.6 ± 2.7, respectively. Although higher temperatures of 80–90 °C produced thinner CNCs with higher aspect ratios, the reduced yield and CrI indicate excessive hydrolysis and partial degradation of crystalline cellulose. Therefore, 70 °C was selected as the optimal reaction temperature for microwave-assisted H_2_SO_4_ hydrolysis.

#### 3.1.4 Effect of irradiation time

The effect of irradiation time on CNC production during microwave-assisted H_2_SO_4_ hydrolysis is shown in Fig. 3A3. Increasing the irradiation time from 10 to 15 min enhanced the CNC yield from 11.3 to 49.0%, while the CrI increased from 75.2 to 86.4%, indicating more effective hydrolysis of amorphous cellulose regions. However, prolonging the irradiation time to 20 and 25 min reduced the yield to 38.8 and 41.4%, respectively, with CrI values of 82.2–82.9%. This decrease is likely attributed to excessive hydrolysis and partial degradation of cellulose at prolonged irradiation times. TEM analysis further confirmed the irradiation time-dependent morphology of the obtained CNCs (Fig. 4C1–C4). The CNCs prepared at 10, 15, 20, and 25 min exhibited average lengths of 311.4 ± 149.6, 109.5 ± 35.5, 130.4 ± 43.9, and 105.9 ± 24.8 nm, respectively, with corresponding diameters of 36.8 ± 10.7, 24.5 ± 11.1, 22.4 ± 8.6, and 20.3 ± 6.7 nm. The corresponding aspect ratios were 8.6 ± 3.4, 5.3 ± 2.7, 6.4 ± 2.4, and 5.7 ± 2.2, respectively. The CNCs prepared at 15 min exhibited well-defined rod-like morphology and good dispersion, whereas longer irradiation times (20–25 min) led to partial degradation of the nanocrystalline structure. These observations agree well with the yield and CrI results, confirming 15 min as the optimal irradiation time.

#### 3.1.5 Mixed acid (H_2_SO_4_/HNO_3_) hydrolysis

In the H_2_SO_4_/HNO_3_ mixed-acid system, cellulose hydrolysis is proposed to proceed through a combination of proton-mediated glycosidic bond cleavage and acid-assisted surface functionalization (Fig. 1C). Similar to the single-acid system, H_2_SO_4_ provides abundant H^+^ species that protonate the glycosidic oxygen atoms in β(1→4)-glycosidic linkages, weakening the C–O–C bonds and promoting chain scission, particularly within the amorphous regions of cellulose. The incorporation of HNO_3_ further increases the acidity and oxidative character of the hydrolyzing medium,^47,48^ which may facilitate fiber swelling, acid penetration,^49,50^ and disruption of more accessible cellulose domains. Under microwave irradiation, the highly polar and ionic H_2_SO_4_/HNO_3_ medium can efficiently convert electromagnetic energy into heat through dipolar polarization and ionic conduction,^51^ resulting in rapid volumetric heating and enhanced cellulose–acid interactions. In addition to the sulfate half-ester groups introduced by H_2_SO_4_ (Fig. 1D.2), the presence of HNO_3_ may promote limited oxidation of accessible cellulose hydroxyl groups (–OH), leading to the formation of carboxyl functionalities (–COOH). After deprotonation, these groups can contribute negatively charged carboxylate species (–COO^−^), which, together with sulfate groups, enhance electrostatic repulsion among CNC particles and improve colloidal stability.

#### 3.1.6 Effect of H_2_SO_4_/HNO_3_ concentration

**Fig. 5:**
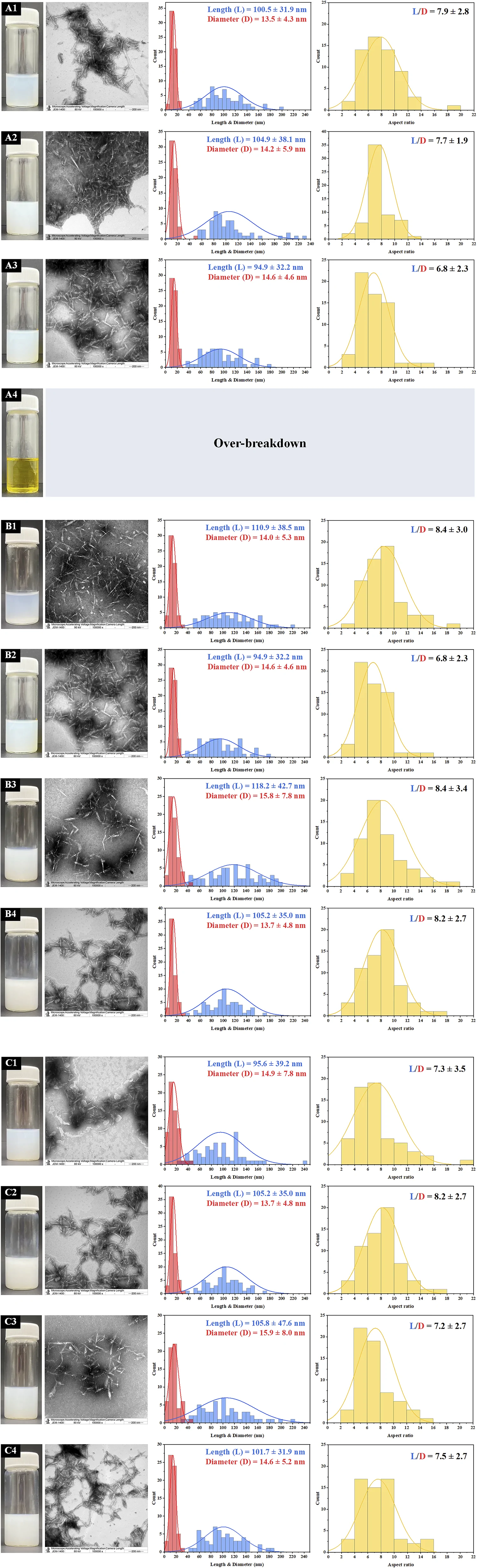
TEM characterization of CNC prepared by microwave-assisted H_2_SO_4_/HNO_3_ hydrolysis under different H_2_SO_4_/HNO_3_ concentration ratios: (A1) 30 : 5% v/v, (A2) 30 : 10% v/v, (A3) 30 : 20% v/v, and (A4) 30 : 30% v/v. The figure includes TEM images, inset digital photographs, diameter-distribution histograms, and aspect ratio analysis. TEM images of CNC prepared by microwave-assisted H_2_SO_4_/HNO_3_ hydrolysis under different hydrolysis temperature: (B1) 60 °C, (B2) 70 °C, (B3) 80 °C, and (B4) 90 °C. The figure includes TEM images, inset digital photographs, diameter-distribution histograms, and aspect ratio analysis. TEM images of CNC prepared by microwave-assisted H_2_SO_4_/HNO_3_ hydrolysis under different hydrolysis times: (C1) 10 min, (C2) 15 min, (C3) 20 min, and (C4) 25 min. The figure includes TEM images, inset digital photographs, diameter-distribution histograms, and aspect ratio analysis.

The influence of the H_2_SO_4_/HNO_3_ concentration ratio on CNC production during microwave-assisted mixed acid hydrolysis shown in Fig. 3B1. At H_2_SO_4_/HNO_3_ ratio of 30 : 5, 30 : 10, and 30 : 20% v/v, the CNC yield was 5.8, 23.3, and 16.4%, respectively, while the corresponding CrI values were 82.0, 85.7, and 87.4%, respectively. Increasing the HNO_3_ concentration from 5 to 10% substantially improved the CNC yield while maintaining high crystallinity, indicating more effective removal of amorphous cellulose regions. However, further increasing the HNO_3_ content to 20% reduced the yield, although the CrI slightly increased, suggesting that stronger mixed-acid conditions promoted excessive hydrolysis and partial dissolution of cellulose fragments. At the highest H_2_SO_4_/HNO_3_ ratio of 30 : 30% v/v, severe over-breakdown occurred, and no recoverable CNCs were obtained. TEM analysis further confirmed the concentration-dependent morphology of CNCs prepared using mixed-acid system (Fig. 5A1–A4). At H_2_SO_4_/HNO_3_ ratios of 30 : 5, 30 : 10, and 30 : 20% v/v, the obtained CNCs exhibited rod-like nanocrystalline morphology with average lengths of 100.5 ± 31.9, 104.9 ± 38.1, and 94.9 ± 32.2 nm, respectively, and corresponding diameters of 13.5 ± 4.3, 14.2 ± 5.9, and 14.6 ± 4.6 nm. The aspect ratios were 7.9 ± 2.8, 7.7 ± 1.9, and 6.8 ± 2.3, respectively. The 30 : 10% v/v condition produced well-dispersed rod-like CNCs with relatively uniform morphology, consistent with its highest CNC yield and high CrI. In contrast, increasing the HNO_3_ content to 20% slightly reduced the aspect ratio and CNC yield, suggesting stronger hydrolysis and partial degradation of nanocrystalline fragments. At 30 : 30% v/v, severe over-breakdown was observed, and no measurable CNC morphology could be obtained, confirming that excessive mixed-acid concentration was detrimental to CNC recovery.

#### 3.1.7 Effect of reaction temperature

The effect of reaction temperature on CNC production during microwave-assisted H_2_SO_4_/HNO_3_ hydrolysis is shown in Fig. 3B2. When the temperature increased from 60 to 90 °C, the CNC yield gradually improved from 3.1 to 21.8%, whereas the CrI remained high within the range of 80.7–88.2%. The low yield at 60 °C suggests that the thermal energy was insufficient to promote effective acid diffusion and cleavage of amorphous cellulose. Raising the temperature to 70–90 °C enhanced cellulose disintegration, probably due to accelerated proton mobility, improved swelling of cellulose fibers, and stronger acid–cellulose interactions under microwave irradiation. Notably, the CrI was maintained above 88.2% at higher temperatures, indicating that the mixed-acid hydrolysis preferentially removed amorphous domains while largely preserving crystalline regions. TEM analysis further confirmed the formation of rod-like CNCs at all tested temperatures (Fig. 5B1–B4). The CNC prepared at 60, 70, 80, and 90 °C exhibited average lengths of 110.9 ± 38.5, 94.9 ± 32.2, 118.2 ± 42.7, and 105.2 ± 35.0 nm, respectively, with corresponding diameters of 14.0 ± 5.3, 14.6 ± 4.6, 15.8 ± 7.8, and 13.7 ± 4.8 nm. The corresponding aspect ratios were 8.4 ± 3.0, 6.8 ± 2.3, 8.4 ± 3.4, and 8.2 ± 2.7, respectively. Although all samples retained rod-like nanoscale structure morphology, 90 °C provided the highest CNC yield while maintaining high crystallinity and a suitable aspect ratio. Therefore, 90 °C was selected as the suitable reaction temperature for microwave-assisted H_2_SO_4_/HNO_3_ hydrolysis.

#### 3.1.8 Effect of irradiation time

The effect of irradiation time on CNC production during microwave-assisted H_2_SO_4_/HNO_3_ hydrolysis is shown in Fig. 3B3. Increasing the irradiation time from 10 to 20 min enhanced the CNC yield from 16.4 to 47.7% while the CrI increased from 80.6 to 89.2%, indicating that longer microwave exposure promoted more effective acid diffusion and hydrolytic removal of amorphous cellulose regions. However, extending the irradiation time to 25 min reduced the CNC yield to 28.2%, although the CrI remained high at 86.4%. This decrease suggests that excessive irradiation time promotes over-hydrolysis and partial dissociation of cellulose fragments. TEM analysis further confirmed the formation of rod-like CNCs at all irradiation times (Fig. 5C1–C4). The CNCs prepared at 10, 15, 20, and 25 min exhibited average lengths of 95.6 ± 39.2, 105.2 ± 35.0, 105.8 ± 47.6, and 101.7 ± 31.9 nm, respectively, with corresponding diameters of 14.9 ± 7.8, 13.7 ± 4.8, 15.9 ± 8.0, and 14.6 ± 5.2 nm. The corresponding aspect ratios were 7.3 ± 3.5, 8.2 ± 2.7, 7.2 ± 2.7, and 7.5 ± 2.7, respectively. Although all samples retained rod-like nanoscale morphology, the 20 min condition provided the highest CNC yield and CrI while maintaining an appropriate aspect ratio. Therefore, 20 min was selected as the optimal irradiation time for microwave-assisted H_2_SO_4_/HNO_3_ hydrolysis.

#### 3.1.9 Conventional heat H_2_SO_4_ hydrolysis

**Fig. 6:**
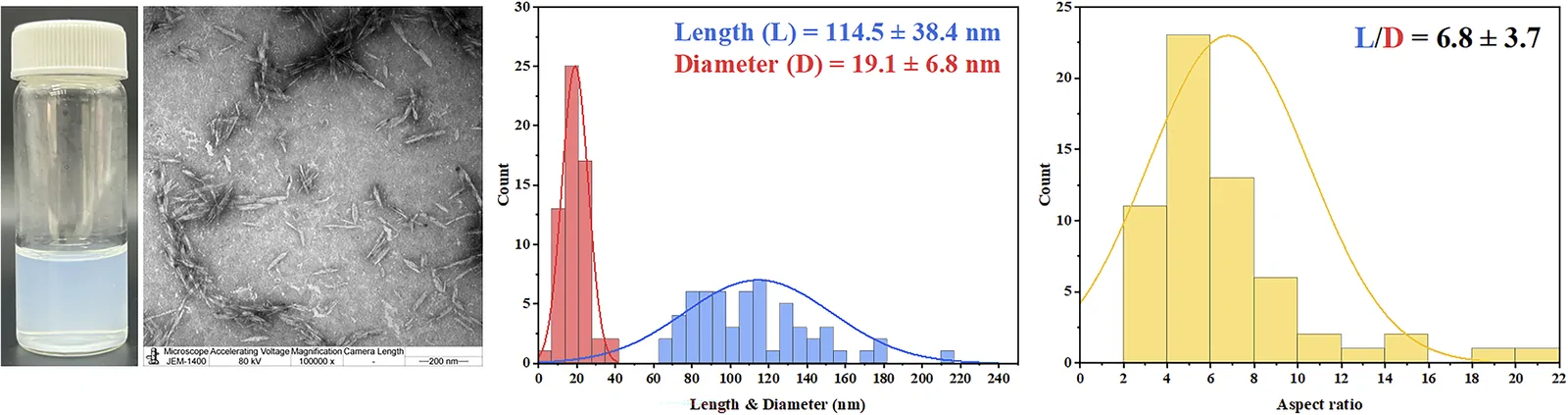
TEM images of CNC prepared by conventional H_2_SO_4_ hydrolysis, with inset digital photographs, diameter-distribution histograms, and aspect ratio analysis.

Conventional H_2_SO_4_ hydrolysis was performed as a comparative control to evaluate the effect of microwave irradiation. As shown in Fig. 3C, conventional heating at 50% H_2_SO_4_ produced CNCs with a CrI of 82.1% and a yield of 12.9%. TEM analysis confirmed the formation of rod-like CNCs (Fig. 6), with an average length of 114.5 ± 38.4 nm, diameter of 19.1 ± 6.8 nm, and aspect ratio of 6.8 ± 3.7.

### 3.2 Physicochemical characterization of CNCs

Based on the optimization results, CNCs prepared under the best conditions were selected for comparative characterization. The optimized microwave-assisted H_2_SO_4_ hydrolysis was performed using 30% H_2_SO_4_ at 70 °C for 15 min, whereas the optimized microwave-assisted H_2_SO_4_/HNO_3_ hydrolysis used an H_2_SO_4_/HNO_3_ ratio of 30 : 20% v/v at 90 °C for 20 min. The resulting CNCs were comparatively evaluated against pristine cotton, alkali-treated cotton, and conventional H_2_SO_4_-hydrolyzed CNCs using XRD, FT-IR, solid-state ^13^C NMR, TEM, XPS, TGA/DTG, and zeta potential analyses to clarify their structural, chemical, thermal, surface, and colloidal characteristics.

#### 3.2.1 XRD analysis

**Fig. 7:**
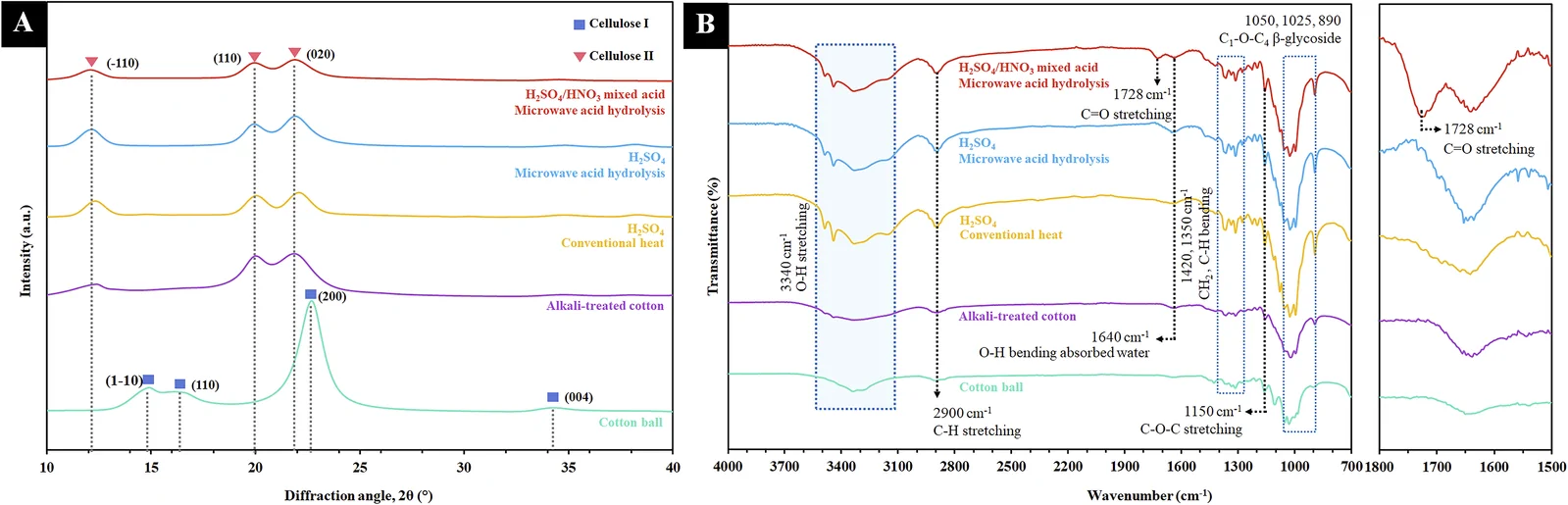
(A) XRD patterns, (B) FT-IR spectra of cotton, alkali-treated cotton, and CNCs prepared by microwave-assisted H_2_SO_4_ hydrolysis, microwave-assisted H_2_SO_4_/HNO_3_ hydrolysis, and conventional H_2_SO_4_ hydrolysis.

The crystalline structures of pristine cotton, alkali-treated cotton, and CNCs prepared by different hydrolysis routes were examined by XRD (Fig. 7A). Pristine cotton showed the typical diffraction pattern of cellulose I, with characteristic peaks at 2*θ* of 14.8°, 16.5°, 22.7°, and 34.5°, corresponding to the (1–10), (110), (200), and (004) crystallographic planes, respectively. These reflections confirm the native cellulose I crystalline polymorph of cotton, which is consistent with the typical crystalline structure reported for cotton-derived cellulose.^52,53^ After alkaline pretreatment, the XRD pattern of alkali-treated cotton exhibited noticeable changes in peak position and intensity. The main reflections appeared at 2*θ* of 12.1°, 20.1°, and 21.8°, corresponding to the (1–10), (110), and (020) planes of cellulose II, respectively. This structural transition indicates partial mercerization of cellulose induced by concentrated NaOH treatment, accompanied by the removal of waxes, pectin, hemicellulose, and surface impurities.^54,55^

For the microwave-assisted H_2_SO_4_-hydrolyzed CNCs, the XRD pattern showed characteristic cellulose II diffraction peaks at 2*θ* of 12.1°, 20.1°, and 21.8°, corresponding to the (1–10), (110), and (020) crystallographic planes, respectively. Similarly, the microwave-assisted H_2_SO_4_/HNO_3_-hydrolyzed CNCs also exhibited characteristic cellulose II diffraction peaks at 2*θ* of 12.1°, 20.1°, and 21.8°, corresponding to the (1–10), (110), and (020) crystallographic planes, respectively. Compared with alkali-treated cotton, both microwave-assisted H_2_SO_4_ and H_2_SO_4_/HNO_3_-hydrolyzed CNCs retained the characteristic cellulose II diffraction peaks at 2*θ* of 12.1°, 20.1°, and 21.8°, corresponding to the (1–10), (110), and (020) crystallographic planes, respectively. The unchanged peak positions indicate that neither hydrolysis route altered the cellulose II crystalline framework. However, the sharper and more intense peaks, especially at 2*θ* = 21.8°, reflect the preferential removal of amorphous cellulose regions and enrichment of crystalline domains. The comparable diffraction patterns of the two microwave-assisted CNCs suggest that both single-acid and mixed-acid systems effectively preserved the ordered cellulose structure during CNC isolation.

In contrast, conventional H_2_SO_4_-hydrolyzed CNCs also retained the characteristic cellulose II diffraction peaks at 2*θ* of 12.1°, 20.1°, and 21.8°, corresponding to the (1–10), (110), and (020) planes, respectively. However, the diffraction peaks were less intense and less sharp than those of the microwave-assisted CNCs, suggesting less efficient enrichment of crystalline domains. This may be attributed to slower heat transfer and less uniform acid–cellulose interactions under conventional heating, which limited the selective removal of amorphous cellulose regions.

#### 3.2.2 FT-IR analysis

FT-IR analysis was performed to identify the functional groups and chemical changes of cotton, alkali-treated cotton, and CNCs prepared by different hydrolysis routes (Fig. 7B). Pristine cotton exhibited typical cellulose absorption bands, including a broad O–H stretching band around 3340 cm^−1^, C–H stretching at 2900 cm^−1^, and C–O–C stretching of the β-glycosidic linkage at 1150 cm^−1^. After alkaline pretreatment, the main cellulose absorption bands at 3340 cm^−1^ (O–H stretching), 2900 cm^−1^ (C–H stretching), 1150 cm^−1^ (C–O–C stretching), and 1050–890 cm^−1^ (C–O–C and β-glycosidic vibrations) were retained, confirming preservation of the cellulose backbone.

After microwave-assisted acid hydrolysis, the CNC samples still exhibited the characteristic cellulose absorption bands, indicating that the cellulose backbone was preserved during hydrolysis. For microwave-assisted H_2_SO_4_-hydrolyzed CNCs, the bands at 1050, 1025, and 890 cm^−1^ became more pronounced, corresponding to C–O–C vibrations and β-glycosidic linkages of cellulose. This suggests the preferential removal of amorphous cellulose regions and enrichment of crystalline cellulose domains. Notably, microwave-assisted H_2_SO_4_/HNO_3_-hydrolyzed CNCs showed an additional absorption band at 1728 cm^−1^, assigned to C=O stretching of carboxyl groups, suggesting limited oxidation of accessible cellulose hydroxyl groups by HNO_3_.

Conventional H_2_SO_4_-hydrolyzed CNCs also retained the main cellulose absorption bands at 3340 cm^−1^ (O–H stretching), 2900 cm^−1^ (C–H stretching), 1150 cm^−1^ (C–O–C stretching), and 1050–890 cm^−1^ (C–O–C and β-glycosidic vibrations), confirming preservation of the cellulose backbone.

#### 3.2.3 NMR analysis

**Fig. 8:**
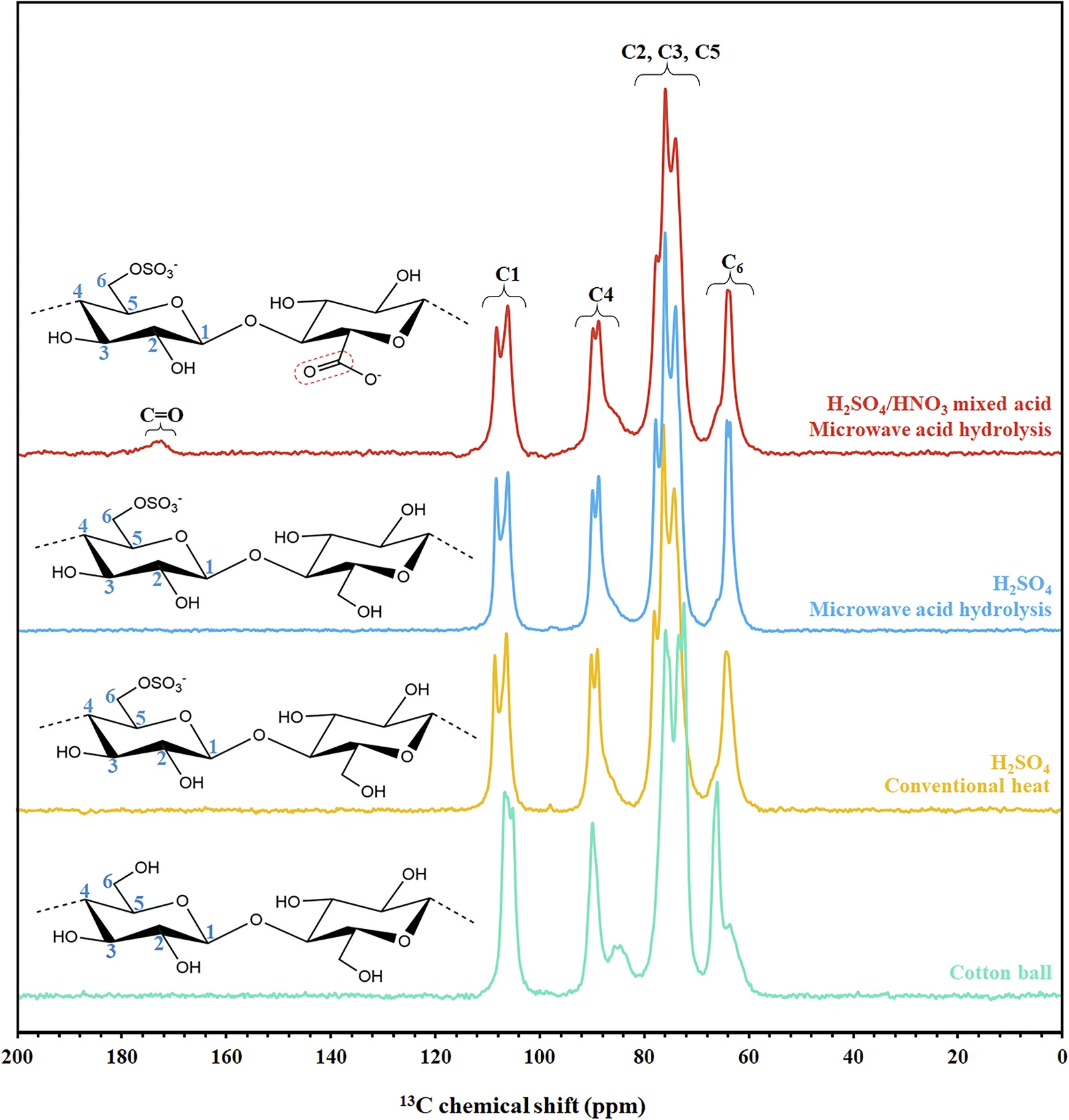
Solid-state ^13^C NMR spectra of cotton, alkali-treated cotton, and CNCs prepared by microwave-assisted H_2_SO_4_ hydrolysis, microwave-assisted H_2_SO_4_/HNO_3_ hydrolysis, and conventional H_2_SO_4_ hydrolysis.

The chemical structure of cotton ball fiber and cellulose nanocrystals (CNCs) synthesized under different hydrolysis conditions was investigated using solid-state ^13^C NMR spectroscopy, as shown in Fig. 8. All samples displayed the characteristic ^13^C resonances of cellulose in the *δ*_C_ 60–110 ppm region, corresponding to the six carbon atoms of the anhydroglucose unit. The resonance at *δ*_C_ 105 ppm was assigned to C-1 of the β-(1→4)-glycosidic linkage, whereas the intense signals at *δ*_C_ 70–80 ppm were attributed to the overlapping resonances of C-2, C-3, and C-5. The signal at *δ*_C_ 60–65 ppm was assigned to C-6 of the primary hydroxymethyl group (–CH_2_OH). The presence of these resonances in all samples confirms that the cellulose molecular framework was preserved after hydrolysis.

The C-4 resonance appeared in the *δ*_C_ 84–90 ppm region, which is sensitive to the ordered and disordered domains of cellulose. The crystalline C-4 signal is typically observed at *δ*_C_ 89 ppm, whereas the amorphous or disordered C-4 contribution appears as a broader shoulder at *δ*_C_ 84 ppm. Compared with the original cotton ball fibers, the CNC samples showed more defined C-4 resonances, indicating preferential removal of amorphous cellulose regions and enrichment of crystalline domains during acid hydrolysis.

Notably, the mixed-assisted H_2_SO_4_/HNO_3_-hydrolyzed CNCs exhibited an additional weak resonance at *δ*_C_ 170–175 ppm, assigned to carbonyl/carboxyl carbon (C=O). This signal suggests the formation of carboxyl functionalities through limited oxidation of accessible cellulose hydroxyl groups, particularly at the C-6 hydroxymethyl position, by HNO_3_ in the mixed-acid system. The introduced –COOH/–COO^−^ groups can provide additional surface functionality, enhancing CNC dispersibility and colloidal stability.

#### 3.2.4 XPS analysis

**Fig. 9:**
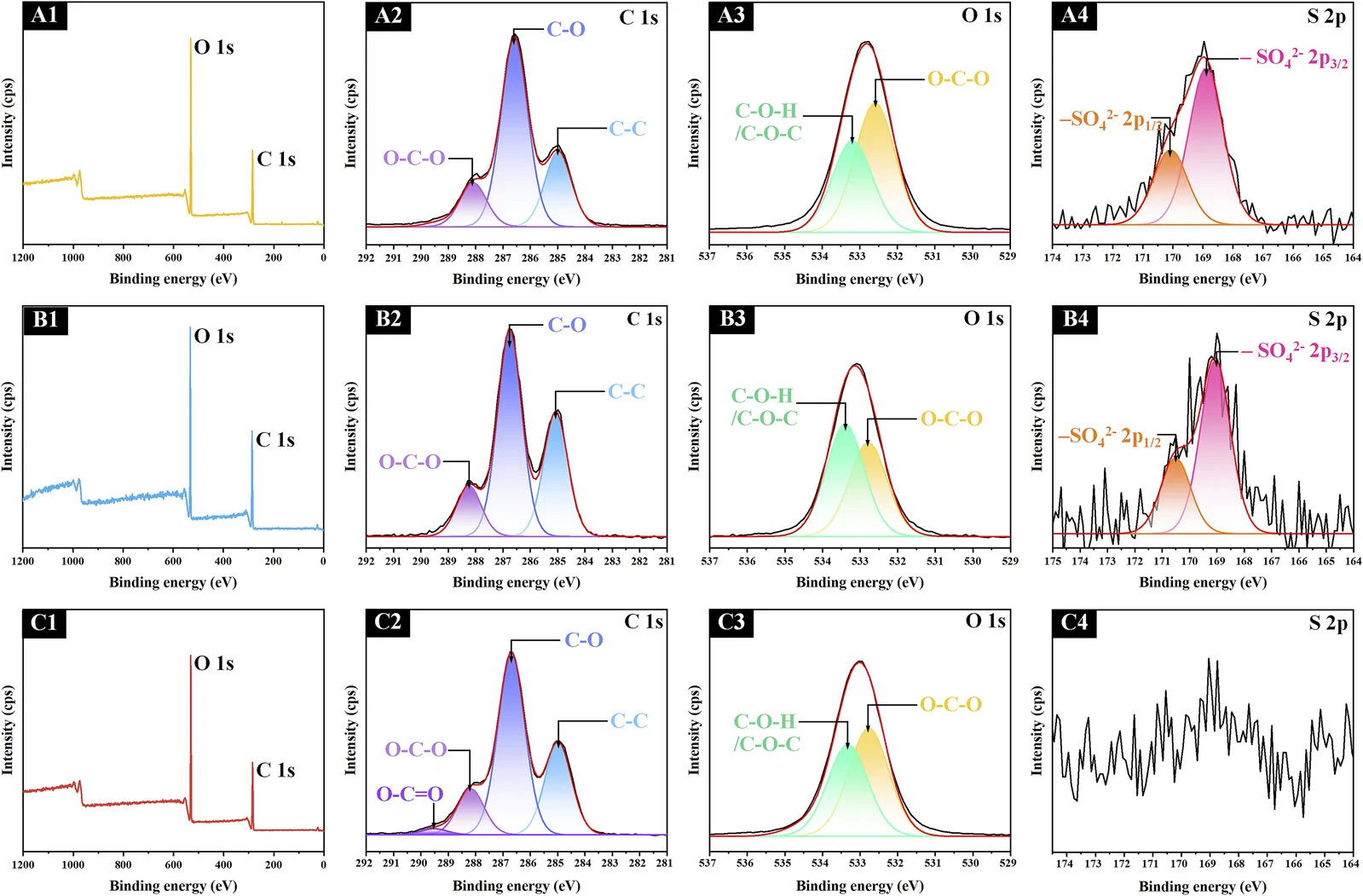
XPS spectra of CNCs prepared by (A) conventional H_2_SO_4_ hydrolysis, (B) microwave-assisted H_2_SO_4_ hydrolysis, and (C) microwave-assisted H_2_SO_4_/HNO_3_ hydrolysis, including (1) survey scan, (2) C 1s, (3) O 1s, and (4) S 2p high-resolution spectra.

XPS analysis was performed to investigate the surface elemental composition and chemical states of CNCs prepared by different hydrolysis routes (Fig. 9). The survey spectra of microwave-assisted H_2_SO_4_-hydrolyzed CNCs, microwave-assisted H_2_SO_4_/HNO_3_-hydrolyzed CNCs, and conventional H_2_SO_4_-hydrolyzed CNCs showed the dominant C 1s and O 1s signals, confirming that the CNC surfaces were mainly composed of carbon and oxygen from the cellulose backbone (Fig. 9A1–C1). The high-resolution C 1s spectra of all CNC samples were deconvoluted into three main components at binding energies of 285.0, 286.7, and 288.2 eV, corresponding to C–C, C–O, and O–C–O environments, respectively (Fig. 9A2 and B2). The O–C=O component at 289.5 eV was more evident for the microwave-assisted H_2_SO_4_/HNO_3_-hydrolyzed CNCs (Fig. 9C2), confirming the partial oxidation of accessible cellulose hydroxyl groups by HNO_3_. The O 1s spectra were fitted into oxygen-containing components at binding energies of 532.8 and 533.3 eV, attributed to O–C–O, C–O–H/C–O–C, respectively (Fig. 9A3–C3). The high-resolution S 2p spectra were further analyzed to verify the presence of sulfate-derived surface groups introduced during H_2_SO_4_ hydrolysis (Fig. 9A4–C4). The microwave-assisted H_2_SO_4_-hydrolyzed CNCs and conventional H_2_SO_4_ hydrolysis exhibited weak sulfate-related S 2p signals at binding energies of 169.0 and 170.5 eV, which can be assigned to S 2p_3/2_ and S 2p_1/2_ of oxidized sulfur species in sulfate half-ester groups, respectively. These sulfate-derived groups originate from the esterification of cellulose hydroxyl groups by H_2_SO_4_ during hydrolysis. In contrast, the mixed-assisted H_2_SO_4_/HNO_3_-hydrolyzed CNCs showed a weaker and less-resolved S 2p response, suggesting a lower amount of detectable sulfate functionalization on the CNC surface.

The bulk elemental composition was further confirmed by CHNS analysis (Table 1). Cotton and alkali-treated cotton were mainly composed of C, H, and O, with no detectable sulfur. After H_2_SO_4_ hydrolysis, sulfur was detected in all CNC samples, confirming the introduction of sulfate-derived groups during acid hydrolysis. The sulfur content was highest for microwave-assisted H_2_SO_4_-hydrolyzed CNCs (1.10 ± 0.03%), followed by microwave-assisted H_2_SO_4_/HNO_3_-hydrolyzed CNCs (0.45 ± 0.04%) and conventional H_2_SO_4_-hydrolyzed CNCs (0.43 ± 0.04%). These results support the XPS observation of sulfate-related S 2p signals and confirm the formation of sulfur-containing functionalities on H_2_SO_4_-hydrolyzed CNCs.

**Table 1:** CHON analysis of cotton ball, alkali-treated cotton, microwave H_2_SO_4_-hydrolyzed CNC, microwave H_2_SO_4_/HNO_3_-hydrolyzed CNC, and conventional H_2_SO_4_-hydrolyzed CNC

Conditions	Carbon (C, %)	Hydrogen (H, %)	Oxygen (O, %)	Sulfur (S, %)
Cotton ball	42.05 ± 0.03	6.10 ± 0.15	51.85 ± 0.17	0.00
Alkali-treated cotton	41.01 ± 0.26	6.45 ± 0.32	52.54 ± 0.13	0.00
Microwave H_2_SO_4_-hydrolyzed CNC	39.61 ± 0.01	6.17 ± 0.22	53.11 ± 0.19	1.10 ± 0.03
Microwave H_2_SO_4_/HNO_3_-hydrolyzed CNC	40.28 ± 0.50	6.36 ± 0.17	52.91 ± 0.29	0.45 ± 0.04
Conventional H_2_SO_4_-hydrolyzed CNC	40.27 ± 0.15	6.21 ± 0.15	53.09 ± 0.09	0.43 ± 0.04

Based on CHONS analysis, the sulfate half-ester contents were calculated to be 0.14, 0.13, and 0.34 mmol g^−1^ for microwave-assisted H_2_SO_4_-hydrolyzed CNCs, microwave-assisted H_2_SO_4_/HNO_3_-hydrolyzed CNCs, and conventional H_2_SO_4_-hydrolyzed CNCs, respectively. The relative O–C=O contributions obtained from C 1s deconvolution were not detected (N.D.), 1.89%, and N.D. for the same samples, respectively. The higher O–C=O contribution of microwave-assisted H_2_SO_4_/HNO_3_-hydrolyzed CNCs supports the formation of carboxyl/carboxylate functionalities through limited oxidation by HNO_3_. Although the O–C=O contribution in the mixed-acid-derived CNCs was relatively low (1.89%), this surface component is important because carboxyl/carboxylate groups are located on the accessible CNC surface. In aqueous suspension, partial deprotonation of –COOH to –COO^−^ can increase the negative surface charge density and enhance electrostatic repulsion between CNC particles. Therefore, even a modest O–C=O contribution may strongly influence the colloidal behavior of CNCs when these groups are exposed and ionizable at the particle surface.

#### 3.2.5 Morphological characteristics

TEM images were used to examine the morphology of CNCs obtained from microwave-assisted acid hydrolysis. CNCs prepared using H_2_SO_4_ hydrolysis exhibited rod-like nanocrystals with partial aggregation, having an average length of 109.5 ± 35.5 nm and diameter of 24.5 ± 11.1 nm (Fig. 2C). In contrast, CNCs produced by mixed H_2_SO_4_/HNO_3_ hydrolysis showed a comparable length of 105.8 ± 47.6 nm but a smaller average diameter of 15.9 ± 8.0 nm (Fig. 2D). The decrease in diameter indicates that the mixed-acid system promoted more effective fibril individualization under microwave irradiation. This improvement may be attributed to the combined hydrolytic action of H_2_SO_4_ and oxidative effect of HNO_3_, which facilitated the removal of amorphous cellulose and weakened interfibrillar interactions.

#### 3.2.6 TGA analysis

**Fig. 10:**
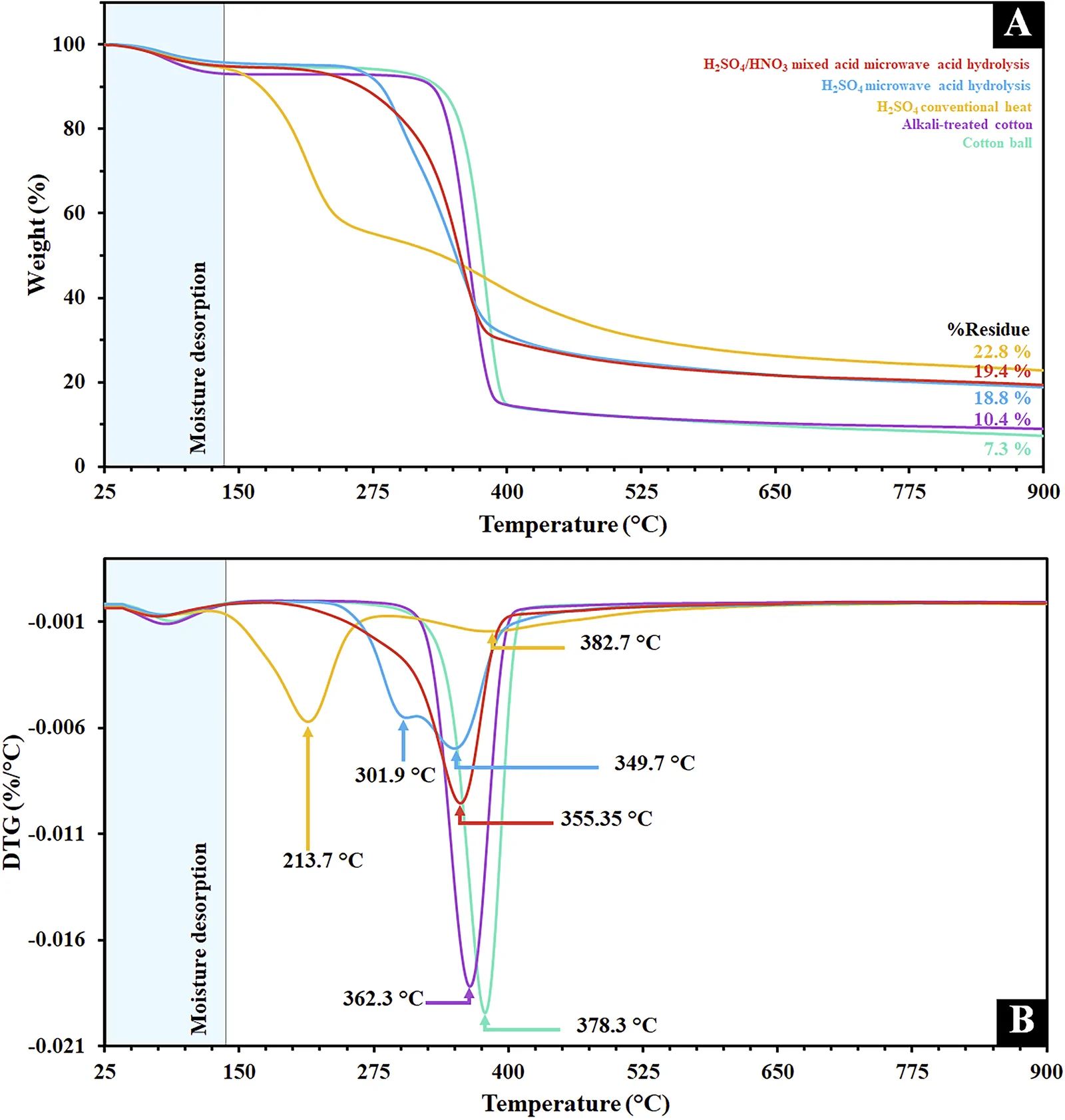
(A) TGA/(B) DTG curves of cotton, alkali-treated cotton, and CNCs prepared by microwave-assisted H_2_SO_4_ hydrolysis, microwave-assisted H_2_SO_4_/HNO_3_ hydrolysis, and conventional H_2_SO_4_ hydrolysis.

The thermal stability of cotton ball, alkali-treated cotton, and CNCs prepared by different hydrolysis routes was evaluated by TGA/DTG analysis, as shown in Fig. 10(A) and (B), and the corresponding thermal parameters are summarized in Table 2. All samples exhibited an initial weight loss below 150 °C, which was attributed to the evaporation of physically adsorbed moisture. Pristine cotton ball exhibited a *T*_5%_ of 320.7 °C and a major degradation peak at 378.3 °C, with the main weight loss occurring between 298 and 438 °C. Alkali-treated cotton showed the highest thermal stability, with a *T*_5%_ of 337.0 °C and a major degradation peak at 362.3 °C. This degradation stage, occurring in the range of 296–423 °C, was associated with depolymerization, dehydration, and decomposition of the cellulose backbone.

**Table 2:** Thermal data of TGA and DTG of cotton ball, alkali-treated cotton, and CNCs prepared by conventional H_2_SO_4_ hydrolysis, microwave-assisted H_2_SO_4_ hydrolysis, and microwave-assisted H_2_SO_4_/HNO_3_ hydrolysis

Materials/method	*T*_5%_ (°C)	1st region			2nd region			3rd region			Residue at 900 °C (%)
		Range (°C)	*T*_max_ (°C)	Weight loss (%)	Range (°C)	*T*_max_ (°C)	Weight loss (%)	Range (°C)	*T*_max_ (°C)	Weight loss (%)	
Cotton ball	320.7	40–145	87.7	5.2	298–438	378.3	80.4	—	—	—	7.3
Alkali-treat cotton	337.0	38–137	76.0	5.8	296–423	362.3	78.5	—	—	—	10.4
Conventional H_2_SO_4_-hydrolyzed CNC	162.4	39–122	73.7	4.6	128–286	213.7	40.4	298–524	382.7	23.1	22.8
Microwave H_2_SO_4_-hydrolyzed CNC	227.9	40–136	80.0	4.2	235–320	301.9	21.4	320–484	349.7	69.2	18.8
Conventional H_2_SO_4_-hydrolyzed CNC	285.7	40–143	76.3	4.8	183–500	355.4	69.8	—	—	—	19.4

Compared with alkali-treated cotton, all CNC samples showed lower thermal stability, as indicated by the decrease in *T*_5%_ from 337.0 °C for alkali-treated cotton to 162.4, 227.9, and 285.7 °C for conventional H_2_SO_4_-hydrolyzed CNCs, microwave-assisted H_2_SO_4_-hydrolyzed CNCs, and microwave-assisted H_2_SO_4_/HNO_3_-hydrolyzed CNCs, respectively. A similar trend was observed in the DTG curves, where the corresponding maximum degradation peaks appeared at 213.7, 301.9/349.7, and 355.4 °C, respectively. The reduced thermal stability of CNCs relative to alkali-treated cotton can be attributed to their nanoscale dimensions, increased surface area, partial depolymerization during acid hydrolysis, and the presence of acid-derived surface functional groups that may catalyze thermal decomposition.

Among the CNC samples, conventional H_2_SO_4_-hydrolyzed CNCs exhibited the lowest *T*_5%_ of 162.4 °C and an early DTG peak at 213.7 °C. This early degradation suggests the formation of low-molecular-weight cellulose fragments and acid-modified surface groups during conventional H_2_SO_4_ hydrolysis, which may accelerate thermal decomposition. In contrast, microwave-assisted H_2_SO_4_-hydrolyzed CNCs showed improved thermal stability, with a *T*_5%_ of 227.9 °C and two degradation regions at 235–320 °C and 320–484 °C. The corresponding DTG peaks appeared at 301.9 and 349.7 °C, corresponding to DTG peaks at 301.9 and 349.7 °C, respectively. The first degradation stage can be associated with the decomposition of less ordered cellulose domains and sulfate-modified surface regions, whereas the second stage corresponds to the degradation of more crystalline cellulose domains. Notably, microwave-assisted H_2_SO_4_/HNO_3_-hydrolyzed CNCs exhibited the highest thermal stability among the CNC samples, with a *T*_5%_ of 285.7 °C and a major DTG peak at 355.4 °C. This enhanced thermal stability can be attributed to the combined effects of structural ordering and controlled surface chemistry. The H_2_SO_4_/HNO_3_ system promoted efficient removal of amorphous cellulose regions, giving the highest CrI value of 89.2% and improved resistance to thermal degradation. Although the O–C=O contribution was relatively low (1.89%), its presence indicates limited surface oxidation and the formation of carboxyl/carboxylate functionalities without substantial disruption of the cellulose framework. In addition, the relatively low sulfate half-ester content of the mixed-acid-derived CNCs (0.13 mmol g^−1^) may reduce the amount of thermally labile sulfate ester groups, which are known to catalyze dehydration and early thermal decomposition. Therefore, the improved thermal behavior of the mixed-acid-derived CNCs is likely associated with the synergistic contribution of higher crystalline ordering, controlled oxygenated surface functionality, and reduced thermally unstable sulfated domains.

The residue contents at 900 °C were 10.4, 22.8, 18.8, and 19.4% for alkali-treated cotton, conventional H_2_SO_4_-hydrolyzed CNCs, microwave-assisted H_2_SO_4_-hydrolyzed CNCs, and microwave-assisted H_2_SO_4_/HNO_3_-hydrolyzed CNCs, respectively. The higher residues of the CNC samples suggest enhanced char formation, likely promoted by acid-derived surface functionalities.

#### 3.2.7 Zeta potential analysis

**Fig. 11:**
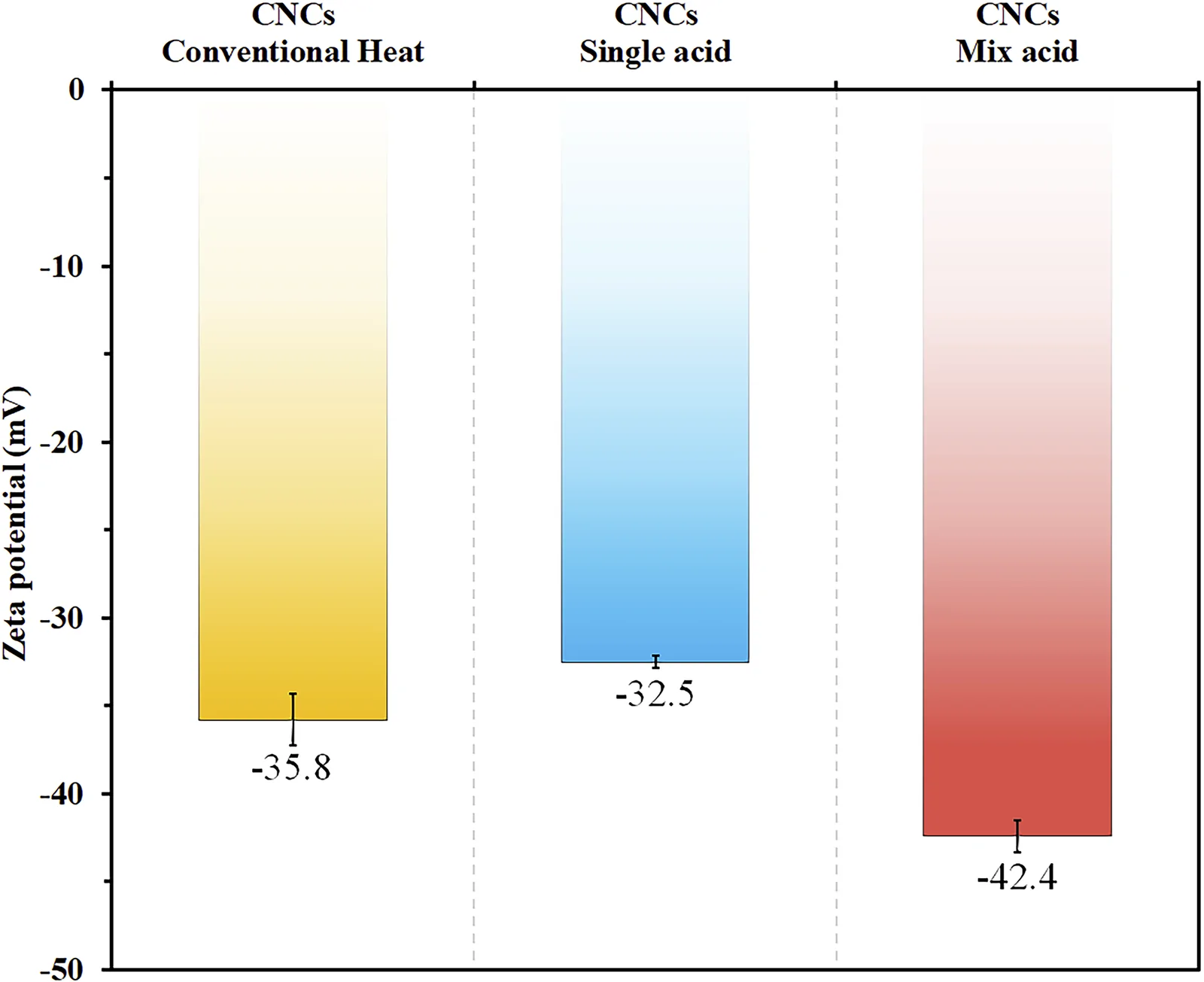
Zeta potential of microwave H_2_SO_4_-hydrolyzed CNC, microwave H_2_SO_4_/HNO_3_-hydrolyzed CNC, and conventional H_2_SO_4_-hydrolyzed CNC.

The zeta potential values of conventional H_2_SO_4_-hydrolyzed CNCs, microwave-assisted H_2_SO_4_-hydrolyzed CNCs, and microwave-assisted H_2_SO_4_/HNO_3_-hydrolyzed CNCs were −35.8 ± 1.5, −32.5 ± 0.4, and −42.4 ± 0.9 mV, respectively (Fig. 11). All CNCs exhibited negative surface charges, indicating the presence of anionic surface groups introduced during acid hydrolysis. The negative charge of H_2_SO_4_-hydrolyzed CNCs is mainly associated with sulfate half-ester groups (–SO_3_^−^), whereas the more negative value of microwave-assisted H_2_SO_4_/HNO_3_-hydrolyzed CNCs suggests the combined contribution of sulfate half-ester (–OSO_3_^−^) and carboxylate (–COO^−^) groups, leading to stronger electrostatic repulsion and improved colloidal stability. This result is consistent with the FT-IR, solid-state ^13^C NMR, and XPS analyses, which indicate the formation of additional carboxyl/carboxylate functionalities in the mixed-acid-derived CNCs. Although the O–C=O contribution obtained from XPS was relatively low (1.89%), the presence of ionizable –COO^−^ groups on the accessible CNC surface can markedly increase the interfacial charge density. Therefore, the improved colloidal stability of the microwave-assisted H_2_SO_4_/HNO_3_-hydrolyzed CNCs is attributed to the combined effect of sulfate half-ester and carboxylate groups rather than the absolute O–C=O contribution alone.

#### 3.2.8 Activation energy

The activation energy of thermal degradation was calculated using the Horowitz–Metzger method. The corresponding plots are shown in Fig. S1, and the calculated values for each degradation stage are summarized in Table S2. For cotton ball and alkali-treated cotton, the main cellulose degradation stage showed activation energy values of 200.3 and 181.9 kJ mol^−1^, respectively. The lower activation energy of alkali-treated cotton may be related to alkali-induced swelling and partial transformation of cellulose I into cellulose II, which reduced interfibrillar interactions and increased cellulose-chain accessibility, thereby lowering the energy barrier for thermal degradation. For conventional H_2_SO_4_-hydrolyzed CNC, two degradation stages were observed, with activation energy values of 51.8 and 40.4 kJ mol^−1^, respectively. For microwave H_2_SO_4_-hydrolyzed CNC, two degradation stages were also observed, with activation energy values of 115.9 and 103.5 kJ mol^−1^, respectively. The higher activation energy in the main degradation stage suggests that microwave-assisted H_2_SO_4_ hydrolysis improved the resistance of CNCs to thermal degradation compared with conventional heating. For microwave H_2_SO_4_/HNO_3_-hydrolyzed CNC, only one main degradation stage was observed, with an activation energy of 82.7 kJ mol^−1^, indicating a more uniform thermal degradation behavior after microwave-assisted mixed-acid hydrolysis.

### 3.3 Comparative analysis and process intensification of microwave-assisted hydrolysis

Table 3 summarizes the key differences among CNCs prepared by microwave-assisted H_2_SO_4_ hydrolysis, microwave-assisted H_2_SO_4_/HNO_3_ hydrolysis, and conventional H_2_SO_4_ hydrolysis. Microwave-assisted H_2_SO_4_ hydrolysis provided the highest CNC yield (49.0%) with a CrI of 86.4% within only 15 min, whereas the mixed-acid H_2_SO_4_/HNO_3_ system produced CNCs with a comparable yield (47.7%) but higher crystallinity (89.2%) and aspect ratio (7.2 ± 2.7) within 20 min. In contrast, conventional H_2_SO_4_ hydrolysis required a much longer reaction time of 120 min but gave a markedly lower yield (12.9%) and lower CrI (82.1%). Statistical analysis was performed using pairwise paired *t*-tests to evaluate whether the differences among hydrolysis methods were statistically meaningful. Three pairwise comparisons were considered: conventional H_2_SO_4_ hydrolysis *versus* microwave-assisted H_2_SO_4_ hydrolysis, conventional H_2_SO_4_ hydrolysis *versus* microwave-assisted H_2_SO_4_/HNO_3_ hydrolysis, and microwave-assisted H_2_SO_4_ hydrolysis *versus* microwave-assisted H_2_SO_4_/HNO_3_ hydrolysis. Statistical significance was determined using the two-tailed *p*-value, with *p* < 0.05 considered significant. As shown in Table S3-1, both microwave-assisted hydrolysis routes produced significantly higher CNC yields than conventional H_2_SO_4_ hydrolysis (*p* < 0.05), while microwave-assisted H_2_SO_4_ hydrolysis also showed a slightly but significantly higher yield than the mixed-acid system (*p* = 0.0126). For crystallinity, the mixed-acid H_2_SO_4_/HNO_3_ system exhibited significantly higher CrI than both conventional H_2_SO_4_ hydrolysis and microwave-assisted H_2_SO_4_ hydrolysis (Table S3-2, *p* < 0.05), supporting more effective amorphous-region removal under the mixed-acid microwave-assisted condition. In terms of morphology, the aspect ratio of the mixed-acid-derived CNCs was significantly higher than that of the microwave-assisted H_2_SO_4_-derived CNCs (*p* = 0.0003), whereas the difference between the mixed-acid and conventional H_2_SO_4_-derived CNCs was not statistically significant (*p* = 0.4347) (Table S3-3). These statistical results support that microwave-assisted hydrolysis significantly improves CNC recovery and crystallinity, while the mixed-acid system provides additional advantages in crystalline-domain enrichment and dimensional control.

**Table 3:** Comparative properties of CNCs prepared by microwave-assisted single-acid H_2_SO_4_, mixed-acid H_2_SO_4_/HNO_3_, and conventional H_2_SO_4_ hydrolysis^a^

Parameter	Microwave-assisted hydrolysis		Conventional heat H_2_SO_4_-hydrolysis
	Single acid (H_2_SO_4_)	Mixed acid (H_2_SO_4_/HNO_3_)	
Optimized condition	30% H_2_SO_4_, 70 °C, 15 min	H_2_SO_4_/HNO_3_ = 30 : 20% v/v, 90 °C, 20 min	50% H_2_SO_4_, 50 °C, 120 min
Main hydrolysis role	Proton-mediated cleavage of amorphous cellulose	Combined proton-mediated hydrolysis and oxidative functionalization	Proton-mediated cleavage of amorphous cellulose
CNC yield (%)	49.0	47.7	12.9
CrI (%)	86.4	89.2	82.1
Average length (nm)	109.5 ± 35.5	105.8 ± 47.6	114.5 ± 38.4
Average diameter (nm)	24.5 ± 11.1	15.9 ± 8.0	19.1 ± 6.8
Aspect ratio (*L*/*D*)	5.3 ± 2.7	7.2 ± 2.7	6.8 ± 3.7
Main surface groups	Sulfate half-ester groups (–OSO_3_^−^)	Sulfate half-ester groups (–OSO_3_^−^), carboxyl/carboxylate functionalities (–COOH/–COO^−^)	Sulfate half-ester groups (–OSO_3_^−^)
Sulfate content (mmol g^−1^)	0.14	0.13	0.34
Relative O–C=O contribution (%)	N.D.	1.89	N.D.
Zeta potential (mV)	−32.5 ± 0.4	−42.4 ± 0.9	−35.8 ± 1.5
*T*_5%_ (°C)	227.9	285.7	162.4
Main DTG peak (°C)	349.7	355.4	213.7

a N.D. = not detected.

The enhanced performance of microwave-assisted hydrolysis can be attributed to rapid volumetric heating and efficient energy transfer within the polar acidic medium. Unlike conventional heating, which relies on heat transfer from the vessel wall to the reaction suspension, microwave irradiation directly interacts with the acid–cellulose system through dipolar polarization and ionic conduction. This heating mode promotes faster temperature elevation, improved acid diffusion, and more effective cleavage of amorphous cellulose regions. Moreover, the acid composition governed the surface chemistry of the resulting CNCs. H_2_SO_4_ mainly introduced sulfate half-ester groups (–OSO_3_^−^), whereas the H_2_SO_4_/HNO_3_ system provided a combined hydrolytic–oxidative pathway, generating both sulfate-derived and carboxyl/carboxylate functionalities (–COOH/–COO^−^). Consequently, the mixed-acid CNCs exhibited the most negative zeta potential (−42.4 ± 0.9 mV) and the highest thermal stability (*T*_5%_ = 285.7 °C), indicating improved colloidal and thermal performance. These findings highlight microwave-assisted mixed-acid hydrolysis as a more controllable and efficient route for producing surface-functionalized CNCs than conventional thermal hydrolysis.

### 3.4 Comparison with previously reported CNC preparation methods

As summarized in Table 4, the microwave-assisted H_2_SO_4_ and H_2_SO_4_/HNO_3_ hydrolysis developed in this work shows clear advantages over previously reported CNC preparation methods in terms of processing time and crystallinity. Most reported approaches, including acid hydrolysis combined with ultrasonication, acetic acid/H_2_SO_4_ hydrolysis, deep eutectic solvent treatment, inorganic oxidation, and TEMPO-mediated oxidation, generally require long reaction times ranging from 1 to 12 h, often together with additional mechanical or oxidative treatment. In contrast, the present method produced CNCs from cotton within only 15 min. Although some previous methods provided higher yields, the CNCs obtained in this work exhibited a relatively high yield of 49.0% and a superior CrI of 86.4%, exceeding those reported for most comparable CNC preparation methods.

**Table 4:** Comparison with previously reported CNC preparation methods

Raw material	Hydrolysis method	Acid/chemical system	Hydrolysis condition	Yield (%)	CrI (%)	Surface functionality	Particle dimension	Thermal stability (*T*_max_, °C)	*ζ*-Potential (mV)	Ref.
Ginger tubers	Acid hydrolysis with ultrasonication	HCl	5 M HCl at 50 °C, 12 h, high ultrasonication processes (600 W, 60 min)	—	48	—	*L*: N.D.	353	—	56
							*D*: 54.3 nm			
Bleached eucalyptus kraft pulp (BEKP)	Acid hydrolysis/heat and stirred	Acetic acid/H_2_SO_4_	Acetic acid (70–90%) with H_2_SO_4_ (5–10%), 80 °C, 1–7 h	60–93	75–81	–OSO_3_^−^	*L*: 150–500 nm	270–367	−30 to −40	57
							*D*: 5–20 nm			
Hardwood bleached kraft pulp	Deep eutectic solvents	Choline chloride/carboxylic acid derivatives	Deep eutectic solvents, 50–100 °C, 3 h	72–88	73–79	–COO^−^	*L*: Microns	—	—	58
							*D*: 10–50 nm			
Wood pulp	Inorganic oxidation	Modified KMnO_4_ with Na_2_H_2_P_2_O_7_	5 g KMnO_4_/Na_2_H_2_P_2_O_7_, room temperature, 1–3 h	72.8	57–66	–COO^−^	*L*: 362–468 nm	339–345	−26 to −38	59
							*D*: 11–18 nm			
Lemon seeds (*Citrus limon*)	Acid hydrolysis and oxidation	H_2_SO_4_, APS and TEMPO oxidation	- 64% (w/w) H_2_SO_4_, 45 °C, 1.5 h	13–52	66–74	–OSO_3_^−^	*L*: 130–170 nm	256–346	−31 to −55	60
			- 1 M APS, 70 °C, 14 h			–COO^−^	*D*: 12–25 nm			
			- TEMPO/NaBr/NaClO, room temperature, 4–5 h							
Okara soybean fiber	Urea solvent and oxidation	NaOH/urea, TEMPO oxidation	8% NaOH/8% urea (w/w), ice bath 30 min and follow by TEMPO oxidation 4–5 h	30	40–68	–COO^−^	*L*: 290–500 nm	280–320	−12 to −33	61
							*D*: N.D.			
Cotton	Microwave-assisted acid hydrolysis	Single-acid H_2_SO_4_	30% H_2_SO_4_, 70 °C, 15 min	49.0	86.4	–OSO_3_^−^	*L*: 109.5 ± 35.5 nm	349.7	−32.5 ± 0.4	This work
							*D*: 24.5 ± 11.1 nm			
		Mixed-acid H_2_SO_4_/HNO_3_	H_2_SO_4_/HNO_3_ = 30 : 20% v/v, 90 °C, 20 min	47.7	89.2	–OSO_3_^−^	*L*: 105.8 ± 47.6 nm	355.4	−42.4 ± 0.9	
						–COO^−^	*D*: 15.9 ± 8.0 nm			

## 4. Conclusions

Microwave-assisted acid hydrolysis was demonstrated as an effective route for isolating CNCs from cotton-derived cellulose. Compared with conventional H_2_SO_4_ hydrolysis, microwave irradiation enhanced acid–cellulose interactions and enabled higher CNC recovery under lower acid concentration and shorter processing time. The optimized microwave-assisted H_2_SO_4_ system produced sulfate-functionalized CNCs with a yield of 49.0%, CrI of 86.4%, aspect ratio of 5.3 ± 2.7, and sulfate content of 0.14 mmol g^−1^. In contrast, the microwave-assisted H_2_SO_4_/HNO_3_ system provided a combined hydrolytic-oxidative pathway, yielding CNCs with a comparable yield of 47.7%, higher CrI of 89.2%, aspect ratio of 7.2 ± 2.7, and additional carboxyl/carboxylate functionalities. Among the prepared CNCs, the mixed-acid microwave-assisted sample exhibited the most balanced properties, including the most negative zeta potential of −42.4 ± 0.9 mV and the highest thermal stability, with a *T*_5%_ of 285.7 °C and a major DTG peak at 355.4 °C. These improvements were attributed to efficient amorphous-region removal and the formation of oxygenated surface groups during H_2_SO_4_/HNO_3_ hydrolysis.

The novelty of this work lies in the systematic comparison of microwave-assisted single-acid and mixed-acid hydrolysis with conventional thermal hydrolysis, demonstrating that microwave-driven mixed-acid treatment can simultaneously intensify CNC production and tailor surface functionality, crystallinity, colloidal stability, and thermal behavior. The shortened reaction time, reduced acid concentration, and improved property control further indicate the broader potential of this approach for industrial-scale CNC production, where process efficiency, resource utilization, and product consistency are critical considerations. These findings highlight microwave-assisted mixed-acid hydrolysis as a controllable and efficient strategy for producing surface-functionalized CNCs with tunable crystallinity, colloidal stability, and thermal performance.

## Author contributions

Chakkaphan Pho-ngernngam: methodology, investigation, data curation, formal analysis, writing – original draft. Piyathida Thaipukdee: methodology, investigation. Nawasit Chotsaeng: formal analysis. Suparat Rukchonlatee: formal analysis. Arjnarong Mathaweesansurn: writing – review & editing. Ekarat Detsri: supervision, conceptualization, data curation, methodology, writing – review & editing, funding acquisition, visualization.

## Conflicts of interest

The authors declare that they have no known competing financial interests or personal relationships that could have appeared to influence the work reported in this paper.

## Supplementary Material

RA-OLF-D6RA05553B-s001

## Data Availability

The datasets generated and analyzed during this study are not publicly available because they are subject to institutional data-sharing policies. Relevant calculation scripts associated with the research process can be made available from the corresponding author upon reasonable request. Supplementary information (SI) is available. See DOI: https://doi.org/10.1039/d6ra05553b.
