# Supplementary material for: Microwave-assisted H2SO4 and H2SO4/HNO3 hydrolysis for process-intensified cellulose nanocrystal production with structural evolution and surface functionalization
Source: RSC Adv. 2026 Sep 16;16(44):55480–503. doi: 10.1039/d6ra05553b (PMC13583438; doi:10.1039/d6ra05553b)
Supplement: Supplementary file 1 [file RA-016-D6RA05553B-s001.pdf]

Supplementary Information (SI) for RSC Advances.
This journal is © The Royal Society of Chemistry 2026

Supporting Information

Microwave-assisted H_2SO_4 and H_2SO_4/HNO_3 hydrolysis for process-intensified cellulose nanocrystal production with structural evolution and surface functionalization

Chakkaphan Pho-ngernngam,[a] Ekarat Detsri,*[ab] Piyathida Thaipukdee,[a] Nawasit Chotsaeng,[ac] Suparat Rukchonlatee,[ad] Arjnarong Mathaweesansurn[ae]

[a]Department of Chemistry, School of Science, King Mongkut's Institute of Technology Ladkrabang, Bangkok 10520, Thailand

[b]Integrated Applied Chemistry Research Unit, School of Science, King Mongkut's Institute of Technology Ladkrabang, Bangkok 10520, Thailand

[c]Advanced Pure and Applied Chemistry Research Unit, School of Science, King Mongkut's Institute of Technology Ladkrabang, Bangkok 10520, Thailand

[d]Polymer Synthesis and Functional Materials Research Unit, School of Science, King Mongkut's Institute of Technology Ladkrabang, Bangkok, Thailand

[e]Applied Analytical Chemistry Research Unit, School of Science, King Mongkut's Institute of Technology Ladkrabang, Bangkok 10520, Thailand

---

Corresponding author:

Ekarat Detsri (E. Detsri, Ph.D)

Department of Chemistry and Integrated Applied Chemistry Research Unit, School of Science, King Mongkut's Institute of Technology Ladkrabang, Bangkok 10520, Thailand

Email address: Ekarat.de@Kmitl.ac.th

https://orcid.org/0000-0001-8627-06

First Author:

Chakkaphan Pho-ngernngam

Department of Chemistry, School of Science, King Mongkut's Institute of Technology Ladkrabang, Bangkok 10520, Thailand

https://orcid.org/0009-0001-4531-894X

Email: 67056007@Kmitl.ac.th

Table Captions

Table		Page
**Table S1**	Summary of representative CNC preparation methods, their advantages, limitations, and key product characteristics.	4
**Fig S1**	Horowitz–Metzger plots for calculating the activation energy (Ea) of thermal degradation for cotton, alkali-treated cotton, and CNCs prepared by different hydrolysis methods.	7
**Table S2**	Activation energy values for each thermal degradation stage of cotton ball, alkali-treated cotton, microwave H_2SO_4-hydrolyzed CNC, microwave H_2SO_4/HNO_3-hydrolyzed CNC, and conventional H_2SO_4-hydrolyzed CNC calculated using the Horowitz–Metzger method.	8
**Table S3-1**	Paired t-test analysis of CNC yield among different hydrolysis methods.	9
**Table S3-2**	Paired t-test analysis of CrI (%) among different hydrolysis methods.	10
**Table S3-3**	Paired t-test analysis of Aspect ratio among different hydrolysis methods.	11

Table S1 Summary of representative CNC preparation methods, their advantages, limitations, and key product characteristics.

CNC preparation method	Main principle	Advantages	Limitations	Key characteristics of obtained CNCs	Ref.
H_2SO_4 hydrolysis	Selective hydrolysis of amorphous cellulose regions by sulfuric acid, with simultaneous introduction of sulfate half-ester groups	High crystallinity; surface functionalization with sulfate groups; improved colloidal stability; effective removal of amorphous regions	Moderate CNC yield; reduced thermal stability due to sulfate groups; requires extensive washing; relatively long hydrolysis time; involves hazardous strong acid	Needle-like or whisker-shaped CNCs; length 100–200 nm; width 10–30 nm; negatively charged surface; CrI up to 89%	25-27
HCl hydrolysis	Selective hydrolysis of amorphous cellulose regions by hydrochloric acid without charged surface-group introduction	High crystallinity; higher thermal stability than H_2SO_4-derived CNCs; relatively simple acid hydrolysis process	Long hydrolysis time; limited surface charge; poor colloidal stability; tendency to aggregate; involves hazardous strong acid	Whisker-shaped CNCs; length 160–250 nm; width 18–23 nm; aspect ratio 10–13; CrI 80–90%	28, 29
Mixed-acid hydrolysis	Combined acid hydrolysis and surface functionalization using mixed acid systems	Selective removal of amorphous regions; tunable surface functionality; improved colloidal stability; high crystallinity; shorter	Requires careful control of acid ratio and concentration to prevent excessive chain scission; may produce shorter particles; involves hazardous strong acids	Whisker-shaped CNCs with length 140–220 nm and width 6–14 nm, or spherical particles with diameter 9–11 nm; negatively charged surface; CrI 84–91%	30, 31

CNC preparation method	Main principle	Advantages	Limitations	Key characteristics of obtained CNCs	Ref.
		hydrolysis time			
Enzymatic treatment	Selective hydrolysis of amorphous cellulose regions using cellulase enzymes	Mild reaction conditions; environmentally friendly process; reduced chemical use	Requires strict pH and temperature control to maintain enzyme activity; slow reaction; limited surface functionalization	Needle-like CNCs; length 1–5 µm; width around 44 nm; CrI 48–67%	32
High-pressure homogenization	Mechanical disintegration of cellulose fibers under high shear and pressure	Acid-free process; avoids neutralization and extensive washing; continuous and scalable production	High energy consumption and production cost; limited surface charge or functional groups; possible aggregation	Nanocellulose particles/fibrils with length 60–320 nm and width 4–14 nm; CrI 50–76%	33
Oxidative processes, e.g., TEMPO-mediated oxidation	Selective oxidation of primary hydroxyl groups on cellulose surfaces to carboxylate groups	Introduces negatively charged carboxylate groups; improves colloidal stability through electrostatic repulsion; enables further functionalization	Efficiency strongly depends on pH and reaction conditions; over-oxidation may decrease yield and crystallinity	Surface-carboxylated nanocellulose with high negative charge and improved aqueous stability; detailed dimensions not reported	34

CNC preparation method	Main principle	Advantages	Limitations	Key characteristics of obtained CNCs	Ref.
Microwave-assisted acid hydrolysis	Acid hydrolysis assisted by microwave irradiation through rapid volumetric heating, dipolar rotation, and ionic conduction	Short reaction time; improved hydrolysis efficiency; reduced chemical consumption; enhanced heating uniformity	Reaction conditions must be carefully optimized to avoid over-hydrolysis or chain scission; yield may decrease under severe conditions	CNCs with tunable morphology, crystallinity, surface charge, and thermal properties depending on acid composition and microwave conditions	37, 38

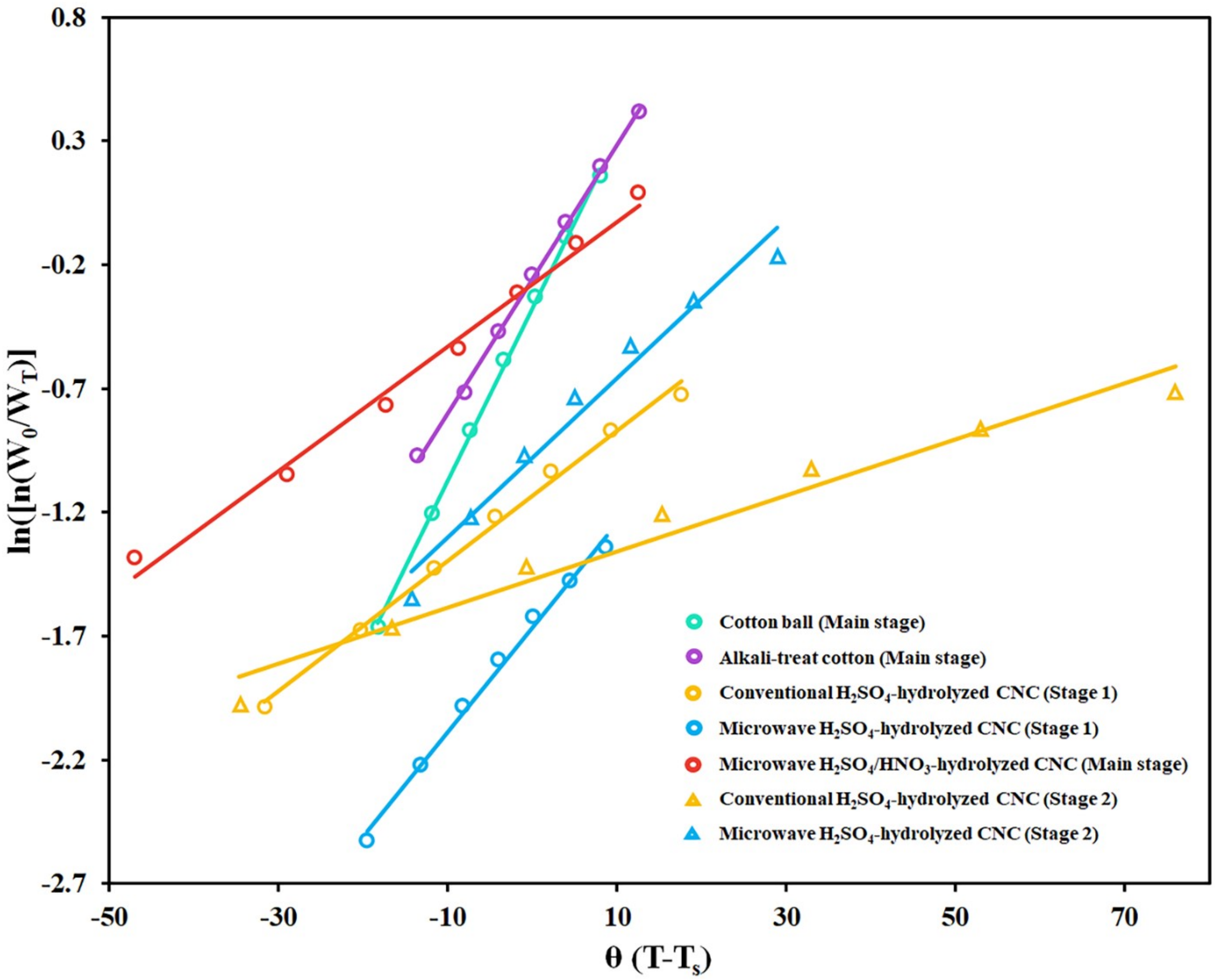

Fig S1 Horowitz–Metzger plots for calculating the activation energy (E_a) of thermal degradation for cotton, alkali-treated cotton, and CNCs prepared by different hydrolysis methods.

Table S2 Activation energy values for each thermal degradation stage of cotton ball, alkali-treated cotton, microwave H_2SO_4-hydrolyzed CNC, microwave H_2SO_4/HNO_3-hydrolyzed CNC, and conventional H_2SO_4-hydrolyzed CNC calculated using the Horowitz–Metzger method.

**Sample**	**Degradation stage**	**Temperature range (°C)**	**T_{max} (°C)**	**Activation energy, (kJ mol^{-1})**
Cotton ball	Main stage	298-438	378.3	200.3
Alkali-treat cotton	Main stage	296-423	362.3	181.9
Conventional H_2SO_4-hydrolyzed CNC	Stage 1	128-286	213.7	51.79
	Stage 2	298-524	382.7	40.4
Microwave H_2SO_4-hydrolyzed CNC	Stage 1	235-320	301.9	115.9
	Stage 2	320-484	349.7	103.5
Microwave H_2SO_4/HNO_3-hydrolyzed CNC	Main stage	183-500	355.4	82.7

Table S3-1 Paired t-test analysis of CNC yield among different hydrolysis methods.

Statistical parameter	Yield (%)					
	**Conventional H_2SO_4 hydrolysis**	**Microwave /Single acid (H_2SO_4)**	**Conventional H_2SO_4 hydrolysis**	**Microwave /Mix acid (H_2SO_4/HNO_3)**	**Microwave /Single acid (H_2SO_4)**	**Microwave /Mix acid (H_2SO_4/HNO_3)**
Mean	12.9460	49.0186	12.9460	47.6500	49.0186	47.6500
Variance	0.9526	0.9671	0.9526	1.3775	0.9671	1.3775
Observations	5	5	5	5	5	5
Pearson correlation	0.9105		0.6524		0.7961	
Hypothesized mean difference	0		0		0	
df	4		4		4	
t Stat	-194.54		-84.905		4.2985	
P(T<=t) one-tail	2.09×10^{-9}		5.77×10^{-8}		0.0063	
t Critical one-tail	2.1318		2.1318		2.1318	
P(T<=t) two-tail	4.19×10^{-9}		1.15×10^{-7}		0.0126	
t Critical two-tail	2.7764		2.7764		2.7764	

Note: Pairwise paired t-tests were performed between the corresponding datasets of each hydrolysis method. Three pairwise comparisons were evaluated: conventional H_2SO_4 hydrolysis vs microwave-assisted H_2SO_4 hydrolysis, conventional H_2SO_4 hydrolysis vs microwave-assisted H_2SO_4/HNO_3 hydrolysis, and microwave-assisted H_2SO_4 hydrolysis vs microwave-assisted H_2SO_4/HNO_3 hydrolysis. Statistical significance was determined using the two-tailed p-value, with $p < 0.05$ considered significant.

Table S3-2 Paired t-test analysis of CrI (%) among different hydrolysis methods.

Statistical parameter	CrI (%)					
	**Conventional H_2SO_4 hydrolysis**	**Microwave /Single acid (H_2SO_4)**	**Conventional H_2SO_4 hydrolysis**	**Microwave /Mix acid (H_2SO_4/HNO_3)**	**Microwave /Single acid (H_2SO_4)**	**Microwave /Mix acid (H_2SO_4/HNO_3)**
Mean	82.0823	86.3598	82.0823	89.2000	86.3598	89.0000
Variance	4.1600	2.1225	4.1600	1.905	2.1225	1.9050
Observations	5	5	5	5	5	5
Pearson correlation	-0.4589		-0.4265		0.2027	
Hypothesized mean difference	0		0		0	
df	4		4		4	
t Stat	-3.1865		-5.4699		-3.5432	
P(T<=t) one-tail	0.0166		0.0027		0.0119	

t Critical one-tail	2.1318		2.1318		2.1318	
P(T<=t) two-tail	0.0333		0.0054		0.0239	
t Critical two-tail	2.7764		2.7764		2.7764	

Note: Pairwise paired t-tests were performed between the corresponding datasets of each hydrolysis method. Three pairwise comparisons were evaluated: conventional H_2SO_4 hydrolysis vs microwave-assisted H_2SO_4 hydrolysis, conventional H_2SO_4 hydrolysis vs microwave-assisted H_2SO_4/HNO_3 hydrolysis, and microwave-assisted H_2SO_4 hydrolysis vs microwave-assisted H_2SO_4/HNO_3 hydrolysis. Statistical significance was determined using the two-tailed p-value, with $p < 0.05$ considered significant.

Table S3-3 Paired t-test analysis of Aspect ratio among different hydrolysis methods.

Statistical parameter	Aspect ratio (L/D)					
	**Conventional H_2SO_4 hydrolysis**	**Microwave /Single acid (H_2SO_4)**	**Conventional H_2SO_4 hydrolysis**	**Microwave /Mix acid (H_2SO_4/HNO_3)**	**Microwave /Single acid (H_2SO_4)**	**Microwave /Mix acid (H_2SO_4/HNO_3)**
Mean	6.8206	5.3035	6.8206	7.2445	5.3035	7.2445
Variance	13.6463	7.5181	13.6463	7.4913	7.5181	7.4913
Observations	60	60	60	60	60	60
Pearson correlation	0.0834		0.1834		-0.0027	
Hypothesized mean difference	0		0		0	

df	59	59	59
t Stat	2.6629	-0.7865	-3.8754
P(T<=t) one-tail	0.0049	0.2174	0.0001
t Critical one-tail	1.6711	1.6711	1.6711
P(T<=t) two-tail	0.0099	0.4347	0.0003
t Critical two-tail	2.0009	2.0009	2.0009

Note: Pairwise paired t-tests were performed between the corresponding datasets of each hydrolysis method. Three pairwise comparisons were evaluated: conventional H_2SO_4 hydrolysis vs microwave-assisted H_2SO_4 hydrolysis, conventional H_2SO_4 hydrolysis vs microwave-assisted H_2SO_4/HNO_3 hydrolysis, and microwave-assisted H_2SO_4 hydrolysis vs microwave-assisted H_2SO_4/HNO_3 hydrolysis. Statistical significance was determined using the two-tailed p-value, with $p < 0.05$ considered significant.
